# From Brewery Waste to Sustainable Aquafeed: Harnessing *Nannochloropsis* Microalgae for Fishmeal‐Free Gilthead Sea Bream Diets

**DOI:** 10.1155/anu/1003936

**Published:** 2026-05-21

**Authors:** Fernando Naya-Català, Ricardo Domingo-Bretón, Qinge Ma, Paul George Holhorea, Álvaro Belenguer, Federico Moroni, Josep Calduch-Giner, Alla Silkina, Danny Van Mullem, Ronald Halim, Jaume Pérez-Sánchez

**Affiliations:** ^1^ Fish Nutrigenomics and Integrative Biology Group, Institute of Aquaculture Torre de la Sal (IATS, CSIC), Ribera de Cabanes, Castellón, Spain; ^2^ UCD Algae Group, School of Biosystems and Food Engineering, University College Dublin, Belfield, Dublin, Ireland, ucd.ie; ^3^ Bioscience Department, Faculty of Science and Engineering, Swansea University, Singleton Park, Swansea, SA2 8PP, UK, swansea.ac.uk; ^4^ LSAqua (Lambers-Seghers NV), Hooglede, Belgium

**Keywords:** aquafeed circular economy, brewery wastewater, gut microbiota, *Nannochloropsis*, transcriptomics, water microbiota

## Abstract

The microalgae *Nannochloropsis oceanica* cultivated from brewery waste was considered a sustainable feed ingredient for gilthead sea bream (*Sparus aurata*), evaluating physiological, molecular and microbial responses. Over 96 days, fish were fed four isoproteic and isolipidic diets: a commercial‐based diet (D1), and three fishmeal‐free diets with increasing *Nannochloropsis* inclusion levels (0%, D2; 2%, D3; 14%, D4). The D4 diet was additionally supplemented with docosahexaenoic acid (DHA)‐rich algae oil instead of fish oil (FO). Growth performance remained almost equal in fish fed all the fishmeal‐free diets with only a slight and final improvement (<4%) in the control D1 fish, although an enhanced expression with a discriminant capacity was reported for hepatic growth‐related markers (*igf1* and *ghr1*) in D4 fish. Gene expression profiling also revealed a down‐regulation of fatty acid synthesis in combination with the up‐regulation of tissue lipid‐uptake genes, mostly linked to the dietary DHA enrichment. Antioxidant capacity was increased in both D3–D4 fish, suggesting a progressive activation of enzymatic and non‐enzymatic defences with the dietary microalgae diets (D3–D4). Moreover, intestinal transcriptomic profiling revealed a tightly regulated immune milieu, with coordinated modulation of pro‐ and anti‐inflammatory pathways indicative of an immune surveillance state induced by dietary microalgae. Water microbiota also shifted with diet composition, indicating a link between feeding strategy and environmental microbial communities. Taking all together, *Nannochloropsis* inclusion, particularly when combined with DHA‐rich microalgae oil, enhances fish health, immune and antioxidant status and beneficial gut microbiota markers. Altogether makes the circularly‐produced *Nannochloropsis oceanica* a valuable resource for the sustainable development of aquaculture feeds.

## 1. Introduction

Population growth, industrialisation and climate change have intensified water scarcity and environmental pollution worldwide, highlighting the urgent need for more sustainable and resilient wastewater treatment solutions. Conventional wastewater management systems, particularly centralised aerobic treatment plants, are not only costly, consuming 3%–4% of electricity in developed countries, but also discharge nutrients insufficiently treated into aquatic ecosystems. This contributes to eutrophication and the ongoing degradation of marine environments [1–4]. Furthermore, such linear treatment approaches perpetuate a resource‐waste paradigm that conflicts with the principles of a circular bioeconomy [5–7]. In response to this, increasing attention is being directed toward wastewater systems designed as resource‐recovery platforms, where valuable products are extracted rather than waste merely mitigated [8–10]. One of the most promising and sustainable solutions for this paradigm shift is the cultivation of microalgae [11, 12]. These photosynthetic microorganisms can sequester excess nutrients from wastewater, remove contaminants and generate high‐value biomass. Biofuels [13, 14], pigments [15] and biofertilizers [16] are among the bioproducts that can be refined after microalgae cultivation in wastewater. Microalgae also represents a valuable source of essential amino acids, polyunsaturated fatty acids (PUFAs), vitamins and natural pigments that can improve fish growth, immunity and survival when added to existing diet formulations to make them more sustainable [17–19]. However, their high fibre content (cellulose and hemicellulose) can reduce digestibility and feed conversion efficiency [20]. Otherwise, the high costs of harvesting, drying and processing microalgal biomass [20] underscore the need to optimize production systems to ensure the profitable use of microalgae in animal nutrition [21]. Despite all this, microalgae have emerged as a valuable aquafeed ingredient, capable of enhancing growth, physiological status, stress resilience and activating immune function, and the health and composition of host‐associated microbiomes in fish and shellfish [18, 22–25].

Compared to other microalgae, species of the genus *Nannochloropsis* offer several advantages that make them especially suitable for aquaculture. Certainly, they are rich in the omega‐3 fatty acid eicosapentaenoic acid (EPA) [26] and exhibit greater stability across diverse salinity, temperature and light conditions, making them less prone to culture crashes [27]. Numerous studies have also demonstrated that the dietary replacement of fishmeal with *Nannochloropsis* spp. improves growth performance, particularly in herbivorous and omnivorous fish [28, 29], while the optimal inclusion level still requires careful adjustment in other farmed fish such as Atlantic salmon [26, 30, 31]. In any case, it is largely assumed that microalgae feeding influences the expression of genes related to growth, immunity, inflammation, metabolism and antioxidant engineering in various tissues, encompassing genes encoding digestive enzymes, inflammatory markers and antioxidant proteins [31–34]. Thanks partly to this transcriptomic antioxidant and immune‐modulatory action, microalgae and DHA addition can also mitigate the adverse effects of a wide range of dietary and environmental stressors, reflected by higher survival rates and improved immunological markers [35]. Moreover, dietary microalgae modulate the intestinal microbiota, typically increasing beta‐diversity and shifting community composition toward beneficial and metabolically active taxa [36–39]. Thus, by providing fermentable substrates, such as complex carbohydrates, microalgae support lactic acid bacteria and other health‐promoting microbes (e.g., *Lactobacillus*), thereby enhancing gut health and resistance to pathogens [40, 41]. Although less studied, the effects on other microbiomes (e.g., gills and skin) are beginning to emerge, supporting the existence of keystone taxa contributing to the stability and functionality of these external microbial ecosystems [42]. However, to our knowledge, the impact of microalgae diets on surrounding‐water microbiomes has not yet been investigated, and advances in sequencing technologies could help address this knowledge gap in an integrative manner [43].

In line with the growing interest in sustainable applications of microalgae within aquaculture systems, an efficient strategy was established to revalorise microalgae harvested from brewery wastewater into sustainable aquafeed [44, 45]. In these studies, *Nannochloropsis oceanica* biomass obtained from cultivation on brewery wastewater was found to be rich in protein (up to 37% DW) and omega‐3 PUFAs in the form of EPA (up to 2.7% DW), indicating their potential as a fishmeal replacement. The application of microalgal biomass cultivated on wastewater as aquafeed, however, has never been evaluated. The bioactive properties of microalgal biomass grown on waste resources are not well elucidated and their effect on fish growth and health is currently unknown, warranting critical investigation. Therefore, the aim of the present study was to investigate the incorporation of *Nannochloropsis* biomass produced from brewery wastewater into fishmeal at different inclusion levels and to evaluate its effects on gilthead sea bream through a comprehensive feeding trial. Growth performance data, blood biochemical parameters, as well as hepatic, head‐kidney and intestinal gene expression associated with metabolism, oxidative stress and immunity, alongside shifts in gut and surrounding‐water microbiota composition were assessed. By integrating physiological, molecular and microbial endpoints, we aimed to elucidate the functional consequences of fishmeal‐free diets and assess the potential of microalgae‐based ingredients to support health and homeostasis in farmed gilthead sea bream and its associated microbial communities. The findings are expected to guide the design and upper inclusion limits of nutritionally balanced, environmentally sustainable microalgae‐based aquafeeds, while providing a comprehensive understanding of the effects of microalgae on farmed fish in general and gilthead sea bream in particular.

## 2. Materials and Methods

### 2.1. Nannochloropsis Biomass

*Nannochloropsis oceanica* (CCAP 849/10) was cultivated in pilot‐scale photobioreactors with a total working volume of 7000 L at Swansea University (UK) on 50:50 v/v mixture of brewery wastewater and standard f medium. The harvested and lyophilised biomass (ca. 3.5 kg DW) was blended with commercially purchased *N. oceanica* powder (ca. 2.9 kg DW) before being dispatched for aquafeed formulations (LSAqua, Belgium).

### 2.2. Animal Ethics

The feeding trial was approved by the Ethics and Animal Welfare Committee of the Institute of Aquaculture Torre de la Sal (IATS), CSIC Ethics Committee (permission 1612/2024) and Generalitat Valenciana (permission 2024‐VSC‐PEA‐0087) to be conducted in the IATS’s aquaculture research infrastructure (code ES120330001055) in accordance with the principles published in the European Animal Directive (2010/63/EU) and Spanish laws (Royal Decree RD53/2013) for the protection of animals used in scientific experimentation.

### 2.3. Experimental Setup and Feeds

One thousand and five hundred juveniles of gilthead sea bream (3–5 g body weight) from a Mediterranean Spanish hatchery (Avramar, Burriana, Spain) were grown‐out in a flow‐through system in a 3000 L tank under natural photoperiod and temperature conditions (40°5′ N; 0°10′ E) for 3 weeks, using a commercial diet (BioMar, Palencia, Spain; EFICO 3053). After this initial acclimation period, 840 fish with an average body weight of 11 g were randomly allocated in twelve 500 L tanks to be fed with one of the four experimental diets for 96 days from April to July 2024 (70 fish per tank; 3 tanks per diet), with water temperature increasing from 17 to 28°C and oxygen concentration remaining above 75% saturation. Water quality was monitored on weekdays for the system, controlling nitrites (0.1 mg/L) and unionised ammonia (0.02 mg/L) concentrations within safe limits, by increasing water‐refreshment level when necessary. Salinity was continuously measured in 38 ± 0.1 ppt (pH ~8), as typical for seawater. All the tested diets were formulated by LSAqua (Hooglede; Belgium) to be isoproteic (42% dry matter) and isolipidic (18% dry matter) feeds with different inclusion levels of fishmeal, fish oil (FO), plant proteins and other alternative ingredients (soya oil [SO], poultry meal, processed animal proteins, single cell proteins and microalgae biomass) to facilitate aquaculture eco‐intensification through increased circularity and resource utilisation (Supporting Information 1: Table S1). Briefly, the control diet (D1) mimicked the formulation of commercial diets with fishmeal (15%), plant proteins (51.5%) and poultry meal (18.3%) as dietary protein sources, and FO and SO as dietary lipids. The positive control (D2) was a fishmeal‐free diet with an increased inclusion level of plant proteins (58.2%), poultry meal (21.9%) and single cell proteins (5%) in combination with a re‐adjusted/increased FO/SO ratio. The other two diets (D3 and D4) remained fishmeal free with higher contents of poultry meal (25%), and increasing inclusion levels of feathermeal hydrolysate (0%–3%) and *Nannochloropsis oceanica* meal (2%–14%) for the replacement of plant proteins. FO and SO persisted as the main sources of dietary oils in the D3 diet. For the most extreme diet formulation (D4), FO was replaced by a DHA‐rich microalgae oil. Such formulation rendered a diet enriched in n‐3 long‐chain PUFAs in comparison to D1–D3 diets (Supporting Information 2: Table S2), resulting the dry matter content (%) of EPA plus docosahexaenoic acid (DHA) close to (0.7%–0.9%, D1–D3) or above (1.80%, D4) the minimum nutrient requirements (0.7%–0.9%) for juveniles of gilthead sea bream [46, 47]. Clear shifts in n‑3:n‑6 PUFAs ratios were also denoted as a consequence of fishmeal and FO replacement. The fish fed with the D1–D3 diets presented low n‑3:n‑6 ratios (0.25–0.27), driven by high linoleic acid (18:2n‑6) inclusion, whereas D4 showed a moderately higher value (0.41) associated with its elevated DHA‐rich oil‐derived content. In this trial, the plant‐based diets did not result in substantial reductions in growth; therefore, a detailed amino acid analysis was not initially prioritised. Nevertheless, we acknowledge the importance of this aspect, and future studies will include a more comprehensive evaluation of dietary amino acid adequacy to better understand the long‐term implications of high plant protein inclusion levels.

### 2.4. Fish Sampling and Sample Collection

At the beginning, intermediate and final biomass sampling points, all fish were individually weighed and measured using a FR‐200 FishReader W (Trovan, Madrid, Spain). At the termination of the feeding trial and following overnight fasting, four fish per tank (12 per diet) were randomly selected and anaesthetised with 0.1 g/L tricaine methanesulfonate (MS 222; Sigma, Saint Louis, MO, USA) to take blood samples from the caudal vessels using heparinised syringes. Blood was centrifuged at 3000 × *g* for 20 min at 4°C, and the retrieved plasma was aliquoted and stored at −20°C until evaluation of metabolite and humoral factors. Fish were then euthanized by cervical section, and the visceral cavity was opened to weigh the liver and viscera, and calculate the hepatosomatic index (HSI) and viscerosomatic index (VSI).

At the end of trial, small tissue portions (∼0.4 cm) of liver, anterior intestine (AI, immediately after the pyloric caeca), and posterior intestine (PI, immediately before the anal ampoule) were taken also for histological analyses. Additionally, small tissue portions (150–200 mg) of liver, AI and head kidney (HK) were excised and collected in RNA later for its storage at −20°C until RNA extraction for gene expression analyses (9 fish per group; 3 fish per tank). The remaining AI (the same 9 fish per group; 3 fish per tank) was opened and gently washed with sterile Hank’s balanced salt solution to remove non‐adherent bacteria. Intestinal mucus was scrapped off using the blunt edge of a sterile scalpel and collected into sterile 1.5 mL tubes. Mucus samples were kept on ice and DNA extraction was performed immediately after sampling (less than 2 h after the initiation of tissue sampling) as described elsewhere [48]. Water samples for microbiota analysis were collected per triplicate directly from the inlet pipe (W‐INT), or from the tanks containing fish (W‐D1, W‐D2, W‐D3 and W‐D4) with sterile glass bottles (1 L). Bacterial biomass was then filtered with a manifold system using mixed cellulose ester filters with a pore size of 0.22 μm. DNA from the water samples was extracted using the DNeasy PowerSoil Pro kit (Qiagen, Hilden, Germany) following the manufacturer’s instructions. The filters were transferred to sterile Petri dishes, cut into small pieces and submitted to mechanical lysis with the ceramic bead tubes provided in the kit, using the FastPrep 24 homogeniser (MP Biomedicals, Irvine, CA, USA) at 6 m/s for 30 s. Of note, this sampling schedule consisted of a single terminal time point, representing a static assessment of dietary responses rather than a longitudinal approach.

### 2.5. Biochemical and Molecular Analyses

Blood haemoglobin was determined using a portable Haemocue portable photometer 201+ (Herli Medical BV, Kontich, Belgium). Plasma glucose was determined using the Invitrogen Glucose Colorimetric Detection Kit (Invitrogen, EIAGLUC). Plasma cortisol levels were determined with an enzyme Immunoassay Kit (Arbor Assays, K003‐H1W) following the manufacturer’s indications. Plasma triglycerides (TGs) were determined using a lipase/glycerol kinase/glycerol‐3‐phosphate oxidase reagent (Cat no. 981786, Thermo Fisher Scientific, Waltham, MA, USA). Total plasma cholesterol was determined using a cholesterol/esterase/cholesterol dehydrogenase reagent (Cat no. 981813, Thermo Fisher Scientific, Waltham, MA, USA). Total antioxidant capacity (TAC) was measured as Trolox activity using a microplate assay kit (Cat no. 709001, Cayman Chemical, Michigan, USA).

For gene expression analyses, RNA from liver, AI and HK was extracted using the MagMAX‐96 total RNA isolation kit (Life Technologies) after tissue homogenisation in TRI reagent following manufacturers’ instructions. RNA quantity and purity was determined by Nanodrop (Thermo Scientific) with absorbance ratios at 260 nm/280 nm of 1.9–2.1. Reverse transcription (RT) of 500 ng of total RNA was performed with random decamers using the High‐Capacity cDNA Archive Kit (Applied Biosystems), and a quantitative PCR was carried out with a CFX96 ConnectTM Real‐Time PCR Detection System (Bio‐Rad, USA), using different 96‐well PCR array layouts designed for the simultaneous profiling of each set of tissue‐selected markers (44–26) in the same plate. The genes comprised in the liver array included markers of the growth hormone (GH) and the insulin‐like growth factor (Igf) axis (9), lipid metabolism (14), oxidative metabolism and energy sensing (12), and antioxidant defence system (9) (Table 1). The arrays of HK and AI included markers of epithelial integrity (11), mucus production (2), nutrient transport (3), cytokines and chemokine‐related proteins (13), T cell and monocyte/macrophage markers (7), pattern recognition receptors (9), immunoglobulins (2) and markers of acute phase response (2) (Table 2). Specific primer pair sequences for liver and HK/AI genes are listed in Supporting Information 3: Table S3 and Supporting Information 4: Table S4, respectively.

**Table 1:** PCR‐array layout for liver ( ^∗^) gene expression profiling.

Function	Gene	Symbol	GenBank
GH/IGF system	Growth hormone receptor‐type 1	*ghr1*	AF438176
	Growth hormone receptor‐type 2	*ghr2*	AY573601
	Insulin‐like growth factor 1	*igf1*	AY996779
	Insulin‐like growth factor 2	*igf2*	AY996778
	Insulin‐like growth factor binding protein 1a	*igfbp1a*	KM522771
	Insulin‐like growth factor binding protein 1b	*igfbp1b*	MH577189
	Insulin‐like growth factor binding protein 2a	*igfbp2a*	MH577190
	Insulin‐like growth factor binding protein 2b	*igfbp2b*	AF377998
	Insulin‐like growth factor binding protein 4	*igfbp4*	KM658998
Lipid metabolism	Elongation of very long chain fatty acids 1	*elovl1*	JX975700
	Elongation of very long chain fatty acids 4	*elovl4*	JX975701
	Elongation of very long chain fatty acids 5	*elovl5*	AY660879
	Elongation of very long chain fatty acids 6	*elovl6*	JX975702
	Fatty acid desaturase 2	*fads2*	AY055749
	Stearoyl‐CoA desaturase 1a	*scd1a*	JQ277703
	Stearoyl‐CoA desaturase 1b	*scd1b*	JQ277704
	Hepatic lipase	*hl*	EU254479
	Lipoprotein lipase	*lpl*	AY495672
	Adipose triglyceride lipase	*atgl*	JX975711
	85 kDa calcium‐independent phospholipase A2	*pla2g6*	JX975708
	Cholesterol 7‐alpha‐monooxygenase	*cyp7a1*	KX122017
	Peroxisome proliferator‐activated receptor α	*pparα*	AY590299
	Peroxisome proliferator‐activated receptor γ	*pparγ*	AY590304
Oxidative metabolism energy sensing	Hypoxia inducible factor 1α	*hif1*α	JQ308830
	Proliferator‐activated receptor γ coactivator 1α	*pgc1*α	JX975264
	Carnitine palmitoyltransferase 1a	*cpt1a*	JQ308822
	Fatty acid binding protein, heart	*hfabp*	JQ308834
	Citrate synthase	*cs*	JX975229
	NADH‐ubiquinone oxidoreductase chain 2	*nd2*	KC217558
	NADH‐ubiquinone oxidoreductase chain 5	*nd5*	KC217559
	Cytochrome c oxidase subunit 1	*cox1*	KC217652
	Cytochrome c oxidase subunit 2	*cox2*	KC217653
	Uncoupling protein 1	*ucp1*	FJ710211
	Sirtuin1	*sirt1*	KF018666
	Sirtuin2	*sirt2*	KF018667
Antioxidant defence	Glutathione peroxidase 1	*gpx1*	DQ524992
	Glutathione peroxidase 4	*gpx4*	AM977818
	Peroxiredoxin 3	*prdx3*	GQ252681
	Peroxiredoxin 5	*prdx5*	GQ252683
	Superoxide dismutase [Cu‐Zn]	*cu-zn-sod/sod1*	JQ308832
	Superoxide dismutase [Mn]	*mn-sod/sod2*	JQ308833
	Glucose‐regulated protein 170 kDa	*grp170*	JQ308821
	Glucose‐regulated protein 94 kDa	*grp94*	JQ308820
	Glucose‐regulated protein 75 kDa	*grp75*	DQ524993

**Table 2:** PCR‐array layout for anterior intestine ( ^∗^) and head kidney (^†^) gene expression profiling.

Function	Gene	Symbol	GenBank
Epithelial integrity	Proliferating cell nuclear antigen	*pcna* ^∗^	KF857335
	Transcription factor HES‐1‐B	*hes1b* ^∗^	KF857344
	Krueppel‐like factor 4	*klf4* ^∗^	KF857346
	Claudin‐12	*cldn12* ^∗^	KF861992
	Claudin‐15	*cldn15* ^∗^	KF861993
	Cadherin‐1	*cdh1* ^∗^	KF861995
	Cadherin‐17	*cdh17* ^∗^	KF861996
	Tight junction protein ZO‐1	*tjp1* ^∗^	KF861994
	Desmoplakin	*dsp* ^∗^	KF861999
	Gap junction Cx32.2 protein	*cx32.2* ^∗^	KF862000
	Coxsackievirus and adenovirus receptor homologue	*cxadr* ^∗^	KF861998
Mucus production	Mucin 2	*muc2* ^∗^	JQ277710
	Mucin 13	*muc13* ^∗^	JQ277713
Nutrient transport	Intestinal‐type alkaline phosphatase	*alpi* ^∗^	KF857309
	Liver type fatty acid‐binding protein	*fabp1* ^∗^	KF857311
	Intestinal fatty acid‐binding protein	*fabp2* ^∗^	KF857310
Cytokines and chemokine‐related proteins	Tumour necrosis factor alpha	*tnfα* ^∗^^†^	AJ413189
	Interleukin‐1 beta	*il1*β ^∗^^†^	AJ419178
	Interleukin‐6	*il6* ^†^	EU244588
	Interleukin‐7	*il7* ^∗^^†^	JX976618
	Interleukin‐8	*il8* ^∗^^†^	JX976619
	Interleukin‐10	*il10* ^∗^^†^	JX976621
	Interleukin‐12 subunit beta	*il12b* ^∗^^†^	JX976624
	Interleukin‐15	*il15* ^∗^^†^	JX976625
	Interleukin‐34	*il34* ^∗^^†^	JX976629
	C–C chemokine receptor type 3	*ccr3* ^∗^^†^	KF857317
	C–C chemokine receptor type 9	*ccr9* ^∗^	KF857318
	C–C chemokine receptor type 11	*ccr11*	KF857319
	C–C chemokine CK8/C–C motif chemokine 20	*ck8/ccl20* ^∗^^†^	GU181393
T cell and monocyte/macrophage markers	Cluster of differentiation 3 zeta chain	*cd3x* ^†^	MF175235
	Cluster of differentiation 4	*cd4-1* ^∗^^†^	AM489485
	Cluster of differentiation 8 alpha	*cd8a* ^†^	EU921630
	Cluster of differentiation 8 beta	*cd8b* ^∗^^†^	KX231275
	Zeta‐chain‐associated protein kinase 70	*zap70* ^†^	MF175239
	Macrophage colony‐stimulating factor 1 receptor 1	*csf1r1* ^∗^^†^	AM050293
	Macrophage mannose receptor 1	*mrc1* ^∗^^†^	KF857326
Pattern recognition receptors (PRRs)	Galectin‐1	*lgals1* ^∗^	KF862003
	Galectin‐8	*lgals8* ^∗^	KF862004
	Toll‐like receptor 2	*tlr2* ^∗^^†^	KF857323
	Toll‐like receptor 5	*tlr5* ^∗^^†^	KF857324
	Toll‐like receptor 9	*tlr9* ^∗^^†^	AY751797
	CD209 antigen‐like protein D	*cd209d* ^∗^	KF857327
	CD302 antigen	*cd302* ^∗^	KF857328
	C‐type lectin domain family 10 member A	*clec10a* ^†^	KF857329
	Fucolectin	*fcl*	KF857331
Immunoglobulins	Immunoglobulin M	*igm* ^∗^^†^	JQ811851
	Immunoglobulin T membrane‐bound form	*igtm* ^∗^^†^	KX599201
	Beta 2 microglobulin	*b2m* ^†^	MF979881
	Caspase 3	*casp3* ^†^	EU722334

Controls of general PCR performance were included on each array, and all the pipetting operations were performed by means of an EpMotion 5070 Liquid Handling Robot (Eppendorf). Briefly, RT reactions were diluted to convenient concentrations and the equivalent of 660 pg of total input RNA was used in a 25 μL volume for each PCR reaction. PCR wells contained a 2 × SYBR Green Master Mix (Bio‐Rad) and specific primers at a final concentration of 0.9 μM were used to obtain amplicons of 50–150 bp in length. The PCR amplification programme consisted of an initial denaturation step at 95°C for 3 min, followed by 40 cycles of denaturation for 15 s at 95°C and annealing/extension for 60 s at 60°C. The efficiency of the PCR reactions was consistently higher than 90%. The specificity of the reactions was verified by melting curve analysis, and linearity of serial dilutions of RT reactions. Gene expression was calculated using the delta–delta Ct method [49]. Expression stability analysis (GeNorm, *M* score = 0.18–0.19) identified β‐actin as the most suitable housekeeping gene for normalisation in all AI, HK and liver tissues. For multigene expression analysis, all values in the liver were referenced to the expression level of *elovl6* in D1 fish with an arbitrary assigned value of 1. In AI and HK, gene expression values were referenced to those of *ccr9* (AI) or *ck8/ccl20* (HK) in D1 fish with an arbitrary assigned value of 1.

### 2.6. Nanopore 16S rRNA Gene Sequencing and Bioinformatic Analysis

The full‐length 16S rRNA gene was amplified using V1–V9 primers and the PCR conditions described elsewhere by Domingo‐Bretón et al. [43]. PCR products were purified using Agencourt AMPure XP beads (Backman Coulter, Brea, CA, United States) with a 0.4 beads/sample ratio. Purified amplicons were quantified using Picogreen assay (Thermo Fisher, Waltham, MA, USA), and visualised in agarose gel (1% w/v TAE buffer) to confirm the amplification of the desired length fragment (~1500 bp). Barcoding sequences and sequencing adapters were attached to purified amplicons by two consecutive ligation reactions using the Native Barcoding kit 96 V14–SQK‐NBD114.96 (Oxford Nanopore Technologies, United Kingdom). Libraries were loaded and sequenced in a R10.4.1 flow‐cell using the PromethION P2 Solo sequencing device. Sequencing data acquisition was managed using MinKNOW v.24.06.16 software. Raw POD5 files were basecalled using Dorado v0.8.2 basecaller (github.com/nanoporetech/dorado) with SUP model (dna_r10.4.1_e8.2_400bps_sup@v5.0.0). Raw data after this procedure is available at NCBI repository (BioProject ID: PRJNA1335540; BioSample IDs: SAMN51991205‐254). Basecalled reads were then demultiplexed and trimmed from barcodes and adapters using the same tool. Resulting BAM files were converted into FASTQ format using samtools v1.10 [50]. Reads were then filtered by quality (Phred q score above 15) and length (1200–1800 bp) using Chopper v.0.8.0 [51]. Quality reports were obtained using NanoPlot v1.42.0 [51]. Filter reads were then taxonomically assigned with minimap2 v2.28‐r1209 (Li, 2021) using SILVA v138.1 as a reference database [52].

### 2.7. Statistics and Visualisations

Statistically significant differences (*p* < 0.05) on growth performance, blood biomarkers and gene expression were assessed by one‐way ANOVA followed by a Holm–Sidak post hoc test, using the SigmaPlot software 14.5 (Systat Software, San Jose, CA, USA). Whenever data normality or homogeneity could not be achieved, nonparametric statistical analysis (Kruskal–Wallis test followed by Dunn’s post‐test) was employed. Statistical analysis of the histological scoring was performed using the non‐parametric Kruskal–Wallis test, followed by Dunn’s post‐test for multiple comparisons. To evaluate potential tank‑level clustering in gene expression and microbiota datasets, we fitted preliminary linear mixed‑effects models including tank as a random effect and calculated the intraclass correlation coefficient (ICC) for each gene and microbiota feature; in all cases ICC values were <0.05, indicating negligible variance attributable to tank. Gene expression patterns were further analysed by partial least‐squares discriminant analysis (PLS‐DA) using EZinfo v3.0 (Umetrics, Umeå, Sweden). Three separate PLS‐DA analyses were conducted, one for each of the analysed tissues, to assess tissue‐specific variations. For a more comprehensive evaluation across tissues, an integrated multi‐tissue PLS‐DA was subsequently performed. The quality of the PLS‐DA models was evaluated by the parameters R2Y(cum) and Q2(cum), which indicate the fit and prediction ability, respectively. Loadings values were analysed to identify the key genes contributing to the group separation along both axes. These loadings of a PLS model typically approximate a continuous distribution centred around a mean value. Thus, loadings exceeding ± 1.96 standard deviations from this mean were considered the most influential variables in the multi‐tissue approach, following the 95% confidence interval criterion. A validation test of the PLS‐DA model consisting of 500 random permutations [53] was performed using the Bioconductor R package ropls [54].

For 16S rRNA gene sequencing data, rarefaction curves, species richness estimates and alpha diversity indexes of microbial communities were obtained using the R package phyloseq [55]. For all analyses, sample depths were normalised by total sum scaling and made proportional to the total sequencing depth [56]. Differences in richness, diversity indexes and phylum and microbial abundance were determined by Kruskal–Wallis test followed by Dunn’s post‐test, with a significance threshold of *p* < 0.05. The beta diversity across groups was tested with permutation multivariate analysis of variance (PERMANOVA), using the non‐parametric method adonis (10,000 random permutations) in the R package vegan [57]. To study the separation between experimental groups, PLS‐DA were performed using EZinfo v.3.0 (Umetrics, Umeå, Sweden), and Hoteling’s T2 statistic was calculated using the same software to detect and report outliers in the model. The contribution of the different taxonomies to the group separation was determined by the minimum variable importance in the projection (VIP) values, where a VIP score ≥1 was considered to be an adequate threshold to determine discriminant taxa in the PLS‐DA model [58]. The quality of the PLS‐DA model was evaluated by the parameters R2Y(cum) and Q2(cum), which indicate fit and prediction ability, respectively. The Bioconductor R package ropls [54] was used to assess whether the supervised model was being over‐fitted (500 random permutations validation test).

## 3. Results

### 3.1. Growth Performance

As shown in Table 3, final body weight and feed intake remained almost equal across D2–D4 dietary groups. However, a slight but significant decrease (*p* < 0.05) in Fulton’s condition factor (CFK) was observed from 2.74 in fish fed the D1–D3 diets to 2.63 in fish of the D4 group. This was related in D4 fish to a reduction of liver weight (*p* = 0.054), which rendered a lower (*p* < 0.01) HSI, despite relatively high variability and the absence of outliers. Fish receiving the control diet (D1) consistently performed well, reaching a specific growth rate (SGR) of 2.09 and a feed conversion ratio (FCR) of 1.03 by the end of the trial, demonstrating that the control diet effectively supported optimal growth and feed efficiency. The growth performance of fish fed the fishmeal‐free diets (D2–D4) remained largely similar among them, disclosing a slight but statistically significant impairment of growth rates (SGR = 2.03–2.05 vs. 2.09; *p* < 0.01) and feed conversion (FCR: 1.08v1.10 vs. 1.03; *p* < 0.05) in comparison to D1 fish.

**Table 3:** Data on growth performance of gilthead sea bream fed the experimental diets (D1, D2, D3 and D4) for 96 days.

Parameter	D1	D2	D3	D4	*p* ^1^
IBW (g)	11.18 ± 0.003	11.18 ± 0.003	11.19 ± 0.003	11.18 ± 0.006	0.330
FBW (g)	84.74 ± 1.26	80.33 ± 1.17	80.73 ± 0.873	80.16 ± 1.102	0.096
FBL (cm)	14.55 ± 0.07	14.21 ± 0.052	14.15 ± 0.078	14.47 ± 0.087	0.052
CFK^2^	2.74 ± 0.00^ab^	2.76 ± 0.036^ab^	2.79 ± 0.033^a^	2.63 ± 0.023^b^	**0.028**
Feed intake (g/fish)	75.6 ± 0.65	75.0 ± 0.63	75.4 ± 1.32	75.9 ± 1.03	0.908
Weight gain (%)	658.2 ± 11.58	618.8 ± 10.40	621.77 ± 7.55	616.7 ± 9.511	0.088
Viscera weight (g)	7.964 ± 0.32	8.415 ± 0.453	7.843 ± 0.352	7.495 ± 0.353	0.384
Liver weight (g)	1.568 ± 0.088	1.642 ± 0.068	1.625 ± 0.097	1.337 ± 0.084	0.054
VSI (%)^3^	9.156 ± 0.339	9.596 ± 0.285	9.527 ± 0.293	8.572 ± 0.207	0.057
HSI (%)^4^	1.80 ± 0.102^ab^	1.88 ± 0.065^a^	1.97 ± 0.095^a^	1.52 ± 0.063^b^	**0.003**
FCR^5^	1.030 ± 0.01^b^	1.083 ± 0.009^a^	1.083 ± 0.009^a^	1.103 ± 0.003^a^	**0.003**
SGR^6^	2.09 ± 0.005^a^	2.04 ± 0.009^b^	2.05 ± 0.007^b^	2.05 ± 0.007^b^	**0.007**

*Note:* Values are the mean ± SEM of triplicated tanks or individual values (biometric data of viscera and liver weight, *n* = 12). Different superscript lowercase letters indicate statistically significant differences among groups (one‐way ANOVA followed by Holm–Sidak post hoc test, *p* ≤ 0.05). Significance of bold values was established in *p* < 0.05.

Abbreviations: FBL, final body length; FBW, final body weight; IBW, initial body weight.

^1^Holm–Sidak post hoc test *p*‐value.

^2^CFK, Fulton’s body condition factor, CFK = 100 × (body weight/standard length^3^).

^3^VSI, viscerosomatic index, VSI = 100 × (viscera weight/fish weight).

^4^HSI, hepatosomatic index, HSI = 100 × (liver weight/fish weight).

^5^FCR, feed conversion rate, FCR = 100 × (dry feed intake/wet weight gain).

^6^SGR, specific growth rate, SGR = 100 × (ln final body weight – ln initial body weight)/days.

### 3.2. Blood Biochemistry

The analysis of blood parameters revealed notable differences in cholesterol, TG and TAC values among the experimental groups (Table 4). Fish fed the D4 diet exhibited higher plasma cholesterol levels (*p* = 0.006) compared to those fed D2 and D3 diets, bringing the values closer to those of D1 fish. Plasma TG levels remained significantly higher (*p* < 0.001) in both D3 and D4 fish in comparison to D2 fish. Regarding TAC, a significant (*p* < 0.001) stepwise increase was observed across all experimental groups, indicating an enhanced TAC in fish as dietary interventions progressed from D1 to D4. No significant differences were observed across all the other analysed blood parameters, including haemoglobin, glucose and cortisol.

**Table 4:** Data on haematology, plasma biochemistry and total antioxidant capacity (TAC) of gilthead sea bream fed the experimental diets for 96 days.

Parameter	D1	D2	D3	D4	*p*‐Value
Haemoglobin (g/dL)	8.25 ± 0.34	8.58 ± 0.15	7.83 ± 0.25	8.87 ± 0.37	0.084
Cholesterol (mg/dL)	263.15 ± 14.46^ab^	226.54 ± 10.87^b^	218.20 ± 15.11^b^	275.37 ± 14.17^a^	**0.006**
Triglycerides (mmol/L)	3.84 ± 0.22^ab^	3.16 ± 0.17^b^	5.02 ± 0.50^a^	5.67 ± 0.49^a^	**<0.001**
Glucose (mg/dL)	86.44 ± 6.78	91.49 ± 5.13	82.56 ± 5.68	97.38 ± .5.35	0.312
Cortisol (ng/mL)	91.71 ± 19.04	127.35 ± 20.95	137.7 ± 26.22	122.881 ± 32.39	0.667
TAC (mM Trolox)	0.46 ± 0.04^d^	0.64 ± 0.04^c^	0.91 ± 0.06^b^	1.24 ± 0.04^a^	**<0.001**

*Note:* Values are the mean ± SEM of nine fish per experimental condition. Different superscript lowercase letters indicate statistically significant differences among groups (one‐way ANOVA followed by Holm–Sidak post hoc test, *p* ≤ 0.05). Significance of bold values was established in *p* < 0.05.

### 3.3. Tissue‐Specific Gene Expression

Up to 20 genes were differentially expressed (*p* < 0.05; one‐way ANOVA) by dietary intervention in the three analysed tissues: 8 in liver, 9 in AI and 3 in HK (Tables 4–6). In the case of liver (Table 5), the expression level of Igf 1 (*igf1*), hepatic lipase (*hl*) and hypoxia‐inducible factor 1α (*hif1*α) were significantly up‐regulated in fish fed the D4 diet. Conversely, a general down‐regulation of lipid metabolism was observed in this group of fish, with a lowered expression of elongases (elongation of very long chain fatty acids 1, *elovl1*; elongation of very long chain fatty acids 6, *elovl6*), desaturases (fatty acid desaturase 2, *fads2*; stearoyl‐CoA desaturase 1a, *scd1a*) and the heart‐type fatty acid‐binding protein (*hfabp*), a small cytosolic protein that functions as the main transporter of long‐chain fatty acids in the cardiomyocytes and hepatocytes. In the AI (Table 6), genes related to the maintenance of the intestinal barrier function, tight junction protein ZO‐1 (*tjp1*), coxsackievirus and adenovirus receptor homologue (*cxadr*) and intestinal‐type alkaline phosphatase (*alpi*) were markedly up‐regulated in D4 fish. Conversely, mucin 2 (*muc2*), a major structural component of the protective mucus layer in gastrointestinal tract, was down‐regulated in this group of fish. Regarding immune function, the expression of interleukin‐1β (*il1b*) become up‐regulated in fish fed the fishmeal‐free diets (D2, D3 and D4). Similarly, interleukin‐8 (*il8*) was significantly up‐regulated in D3 and D2 groups compared to D1, but no significant differences were observed between D4 and the other dietary groups. Additionally, the expression of interleukin‐34 (*il34*) was significantly up‐regulated in D4‐fed fish in comparison to the other three experimental groups. These fish also displayed a higher expression of macrophage colony‐stimulating factor 1 receptor 1 (*csf1r1*), which was statistically significant related to D1 fish. In contrast, the expression of C–C chemokine CK8/C–C motif chemokine 20 (*ck8/ccl20*) was significantly enhanced in D1 fish in comparison to those fed D2. In the HK (Table 7), this feature rendered the down‐regulation of interleukin‐7 (*il7*) in fish fed D2 diet, with a rebound effect in D3 fish that persisted in the D4 fish group. An enhanced gene expression was also found for the immunoglobulin T membrane‐bound form (*igtm*) in D3 and specially D4 fish. Conversely, the expression of caspase 3 (*casp3*) was significantly up‐regulated in D2 fish in comparison to both D1 and D4 fish groups.

**Table 5:** Relative gene expression of liver mRNA transcripts of fish fed the experimental diets.

Gene	D1	D2	D3	D4	*p*‐Value
*ghr1*	0.90 ± 0.19	1.04 ± 0.15	0.84 ± 0.15	1.37 ± 0.22	0.249
*ghr2*	1.18 ± 0.25	1.34 ± 0.19	1.18 ± 0.21	1.43 ± 0.16	0.773
*igf1*	3.65 ± 0.64^b^	4.00 ± 0.51^b^	4.04 ± 0.57^b^	7.51 ± 1.02^a^	**0.002**
*igf2*	1.67 ± 0.40	1.77 ± 0.27	2.09 ± 0.35	1.47 ± 0.27	0.626
*igfbp1a*	0.02 ± 0.00	0.02 ± 0.00	0.02 ± 0.00	0.03 ± 0.01	0.863
*igfbp1b*	0.39 ± 0.18	0.52 ± 0.22	0.51 ± 0.18	0.85 ± 0.31	0.789
*igfbp2a*	0.55 ± 0.12	0.70 ± 0.11	0.70 ± 0.12	0.76 ± 0.11	0.353
*igfbp2b*	0.88 ± 0.16	1.04 ± 0.15	0.98 ± 0.14	1.35 ± 0.15	0.147
*igfbp4*	0.64 ± 0.06	0.57 ± 0.05	0.51 ± 0.05	0.67 ± 0.05	0.147
*elovl1*	4.85 ± 0.64^a^	3.92 ± 0.24^ab^	4.38 ± 0.35^ab^	3.16 ± 0.27^b^	**0.040**
*elovl4*	0.18 ± 0.02	0.17 ± 0.02	0.17 ± 0.02	0.17 ± 0.02	0.982
*elovl5*	2.75 ± 0.43	3.52 ± 0.68	2.73 ± 0.55	5.21 ± 1.12	0.193
*elovl6*	1.10 ± 0.16^a^	1.10 ± 0.17^a^	0.87 ± 0.16^ab^	0.46 ± 0.06^b^	**0.011**
*fads2*	5.47 ± 0.81^a^	4.98 ± 0.65^ab^	5.23 ± 0.74^a^	2.93 ± 0.45^b^	**0.035**
*scd1a*	0.35 ± 0.06^ab^	0.50 ± 0.08^a^	0.33 ± 0.06^ab^	0.16 ± 0.02^b^	**0.004**
*scd1b*	0.49 ± 0.13	0.95 ± 0.25	0.53 ± 0.27	0.51 ± 0.13	0.261
*hl*	4.46 ± 0.49^b^	5.76 ± 0.42^ab^	4.68 ± 0.49^ab^	6.11 ± 0.44^a^	**0.043**
*lpl*	1.85 ± 0.25	2.19 ± 0.19	1.89 ± 0.17	1.87 ± 0.38	0.763
*atgl*	0.16 ± 0.05	0.16 ± 0.04	0.14 ± 0.07	0.27 ± 0.09	0.497
*pla2g6*	0.10 ± 0.01	0.08 ± 0.01	0.08 ± 0.01	0.09 ± 0.01	0.200
*cyp7a1*	0.76 ± 0.15	0.71 ± 0.09	0.62 ± 0.15	0.75 ± 0.12	0.864
*pparα*	1.38 ± 0.20	1.12 ± 0.11	1.10 ± 0.11	1.33 ± 0.05	0.299
*pparγ*	0.40 ± 0.05	0.44 ± 0.04	0.41 ± 0.05	0.56 ± 0.04	0.066
*hif1*α	0.43 ± 0.03^b^	0.44 ± 0.03^ab^	0.43 ± 0.02^b^	0.52 ± 0.02^a^	**0.048**
*pgc1*α	0.05 ± 0.01	0.03 ± 0.01	0.04 ± 0.01	0.04 ± 0.01	0.101
*cpt1a*	0.50 ± 0.04	0.42 ± 0.04	0.51 ± 0.03	0.41 ± 0.05	0.207
*hfabp*	44.34 ± 5.65^a^	36.99 ± 3.58^ab^	38.40 ± 3.26^ab^	25.58 ± 3.29^b^	**0.021**
*cs*	0.59 ± 0.05	0.50 ± 0.03	0.56 ± 0.05	0.62 ± 0.06	0.419
*nd2*	24.13 ± 2.84	20.56 ± 2.30	19.87 ± 1.76	22.65 ± 5.83	0.484
*nd5*	13.38 ± 1.68	10.75 ± 1.07	10.10 ± 0.98	13.56 ± 3.60	0.557
*cox1*	75.10 ± 8.59	59.61 ± 3.17	54.97 ± 3.99	88.90 ± 22.88	0.154
*cox2*	38.54 ± 5.25	29.84 ± 1.75	28.77 ± 2.18	42.46 ± 12.08	0.630
*ucp1*	9.77 ± 1.14	13.69 ± 1.29	10.96 ± 1.41	12.62 ± 0.85	0.116
*sirt1*	0.07 ± 0.01	0.06 ± 0.00	0.06 ± 0.00	0.06 ± 0.01	0.529
*sirt2*	0.15 ± 0.01	0.14 ± 0.01	0.14 ± 0.01	0.16 ± 0.01	0.522
*gpx1*	2.00 ± 0.32	1.29 ± 0.09	1.34 ± 0.08	1.29 ± 0.20	0.098
*gpx4*	6.54 ± 0.68	7.09 ± 0.69	6.47 ± 0.72	7.88 ± 0.47	0.397
*prdx3*	0.49 ± 0.04	0.61 ± 0.06	0.53 ± 0.03	0.66 ± 0.09	0.415
*prdx5*	0.42 ± 0.03	0.46 ± 0.05	0.43 ± 0.04	0.45 ± 0.02	0.912
*cu-zn-sod/sod1*	4.99 ± 0.36	5.33 ± 0.43	5.39 ± 0.53	6.65 ± 0.50	0.079
*mn-sod/sod2*	0.60 ± 0.03	0.64 ± 0.05	0.58 ± 0.03	0.79 ± 0.10	0.419
*grp170*	0.49 ± 0.05	0.45 ± 0.02	0.55 ± 0.06	0.59 ± 0.06	0.288
*grp94*	1.55 ± 0.13	1.67 ± 0.15	1.88 ± 0.30	1.76 ± 0.26	0.904
*grp75*	0.28 ± 0.02	0.24 ± 0.01	0.28 ± 0.03	0.36 ± 0.04	0.092

*Note:* Values are the mean ± SEM of nine fish per experimental condition. All data are in reference to the expression level of *elovl6* of CTRL fish with an arbitrarily assigned value of 1. Different superscript lowercase letters indicate statistically significant differences among groups (one‐way ANOVA followed by Holm–Sidak post hoc test, *p* < 0.05). Significance of bold values was established in *p* < 0.05.

**Table 6:** Relative gene expression of anterior intestine mRNA transcripts of fish fed the experimental diets.

Gene	D1	D2	D3	D4	*p*‐Value
*pcna*	2.91 ± 0.36	3.41 ± 0.57	3.61 ± 0.57	3.12 ± 0.40	0.778
*hes1b*	1.17 ± 0.19	2.11 ± 0.50	1.77 ± 0.26	2.26 ± 0.43	0.182
*klf4*	0.71 ± 0.17	0.70 ± 0.19	0.61 ± 0.12	0.78 ± 0.22	0.832
*cldn12*	0.38 ± 0.06	0.47 ± 0.08	0.40 ± 0.04	0.44 ± 0.05	0.698
*cldn15*	21.02 ± 4.10	30.65 ± 8.41	23.18 ± 4.94	29.66 ± 4.70	0.499
*cdh1*	8.84 ± 1.17	8.53 ± 0.81	8.05 ± 0.70	9.78 ± 1.00	0.570
*cdh17*	31.71 ± 4.63	35.06 ± 5.15	38.70 ± 5.17	43.22 ± 5.58	0.461
*tjp1*	0.21 ± 0.03^b^	0.24 ± 0.04^ab^	0.22 ± 0.02^b^	0.33 ± 0.04^a^	**0.047**
*dsp*	5.06 ± 0.45	4.88 ± 0.36	4.96 ± 0.48	5.17 ± 0.31	0.961
*cx32.2*	85.93 ± 4.89	88.57 ± 9.61	71.50 ± 4.99	98.13 ± 12.41	0.220
*cxadr*	3.04 ± 0.25^b^	2.83 ± 0.18^b^	3.02 ± 0.24^b^	4.46 ± 0.29^a^	**0.003**
*muc2*	77.42 ± 7.72^a^	67.74 ± 3.33^a^	62.62 ± 8.75^ab^	39.11 ± 3.71^b^	**0.001**
*muc13*	35.48 ± 3.97	38.98 ± 2.17	42.63 ± 4.69	44.29 ± 4.06	0.535
*alpi*	66.86 ± 10.20^ab^	53.33 ± 4.08^b^	62.25 ± 10.87^ab^	93.63 ± 11.65^a^	**0.018**
*fabp1*	118.36 ± 13.34	137.37 ± 26.96	125.10 ± 15.77	139.25 ± 7.15	0.302
*fabp2*	538.60 ± 164.54	579.49 ± 98.00	508.51 ± 123.28	840.62 ± 115.13	0.212
*tnfα*	0.12 ± 0.01	0.16 ± 0.01	0.15 ± 0.02	0.15 ± 0.01	0.185
*il1b*	0.05 ± 0.00^b^	0.07 ± 0.01^ab^	0.08 ± 0.01^a^	0.08 ± 0.02^ab^	**0.029**
*il7*	0.33 ± 0.03	0.33 ± 0.03	0.32 ± 0.02	0.34 ± 0.02	0.900
*il8*	0.20 ± 0.03^b^	0.32 ± 0.02^a^	0.38 ± 0.06^a^	0.24 ± 0.03^ab^	**0.021**
*il10*	0.11 ± 0.01	0.12 ± 0.02	0.13 ± 0.02	0.13 ± 0.02	0.808
*il12b*	0.51 ± 0.02	0.52 ± 0.05	0.53 ± 0.08	0.44 ± 0.03	0.446
*il15*	0.62 ± 0.06	0.68 ± 0.06	0.68 ± 0.09	0.75 ± 0.03	0.152
*il34*	0.51 ± 0.02^b^	0.54 ± 0.03^b^	0.53 ± 0.05^b^	0.68 ± 0.02^a^	**0.007**
*ccr3*	0.54 ± 0.05	0.58 ± 0.05	0.56 ± 0.10	0.67 ± 0.08	0.607
*ccr9*	1.12 ± 0.14	1.06 ± 0.11	1.03 ± 0.12	1.16 ± 0.10	0.846
*ccr11*	4.93 ± 0.69	4.53 ± 0.33	3.87 ± 0.60	4.56 ± 0.33	0.524
*ck8/ccl20*	3.82 ± 0.42^a^	2.40 ± 0.65^b^	2.49 ± 0.25^ab^	2.72 ± 0.36^ab^	**0.045**
*cd4-1*	0.29 ± 0.11	0.25 ± 0.03	0.24 ± 0.02	0.27 ± 0.02	0.445
*cd8b*	0.09 ± 0.02	0.07 ± 0.01	0.06 ± 0.01	0.07 ± 0.02	0.498
*csf1r1*	0.10 ± 0.01^b^	0.14 ± 0.01^ab^	0.17 ± 0.03^ab^	0.20 ± 0.02^a^	**0.004**
*mrc1*	0.81 ± 0.06	1.02 ± 0.09	0.92 ± 0.14	1.01 ± 0.08	0.278
*lgals1*	11.60 ± 1.48	12.43 ± 1.43	11.61 ± 1.03	12.47 ± 1.66	0.949
*lgals8*	2.41 ± 0.20	2.88 ± 0.16	3.37 ± 0.43	3.23 ± 0.29	0.091
*tlr2*	0.66 ± 0.07	0.77 ± 0.12	0.82 ± 0.15	0.73 ± 0.05	0.809
*tlr5*	0.04 ± 0.01	0.03 ± 0.00	0.03 ± 0.00	0.04 ± 0.00	0.660
*tlr9*	0.05 ± 0.01	0.05 ± 0.01	0.04 ± 0.00	0.04 ± 0.00	0.986
*cd209d*	0.14 ± 0.02	0.14 ± 0.01	0.14 ± 0.02	0.13 ± 0.01	0.851
*cd302*	9.23 ± 0.76	8.71 ± 0.31	8.24 ± 0.72	9.38 ± 0.38	0.337
*fcl*	5.48 ± 2.29	3.02 ± 0.56	3.79 ± 1.78	3.98 ± 1.91	0.560
*igm*	2.03 ± 0.51	6.60 ± 2.43	6.78 ± 1.97	6.28 ± 1.18	0.071
*igtm*	0.46 ± 0.11	0.87 ± 0.17	0.88 ± 0.32	0.80 ± 0.28	0.444

*Note:* Values are the mean ± SEM of nine fish per experimental condition. All data are in reference to the expression values of *ccr9* of CTRL fish with an arbitrarily assigned value of 1. Different superscript lowercase letters indicate statistically significant differences among groups (one‐way ANOVA followed by Holm–Sidak post hoc test, *p* < 0.05). Significance of bold values was established in *p* < 0.05.

**Table 7:** Relative gene expression of head kidney mRNA transcripts of fish fed the experimental diets.

Gene	D1	D2	D3	D4	*p*‐Value
*tnfα*	0.23 ± 0.02	0.25 ± 0.02	0.25 ± 0.03	0.26 ± 0.03	0.827
*il1b*	0.12 ± 0.02	0.13 ± 0.02	0.18 ± 0.02	0.18 ± 0.05	0.261
*il6*	0.02 ± 0.00	0.02 ± 0.00	0.03 ± 0.01	0.02 ± 0.00	0.564
*il7*	0.79 ± 0.07^ab^	0.64 ± 0.04^b^	0.99 ± 0.11^a^	0.87 ± 0.08^ab^	**0.024**
*il8*	0.09 ± 0.01	0.12 ± 0.03	0.12 ± 0.02	0.13 ± 0.03	0.601
*il10*	0.63 ± 0.07	0.73 ± 0.08	0.91 ± 0.17	0.65 ± 0.09	0.628
*il12*	0.05 ± 0.01	0.06 ± 0.01	0.06 ± 0.01	0.06 ± 0.02	0.700
*il15*	0.59 ± 0.06	0.74 ± 0.11	0.72 ± 0.15	0.61 ± 0.14	0.759
*il34*	1.34 ± 0.11	1.10 ± 0.11	1.20 ± 0.22	0.98 ± 0.12	0.438
*ccr3*	6.95 ± 0.42	7.12 ± 0.62	6.41 ± 0.50	6.67 ± 0.48	0.771
*ck8/ccl20*	1.04 ± 0.10	1.27 ± 0.21	1.18 ± 0.21	1.07 ± 0.27	0.489
*cd3x*	2.60 ± 0.23	3.30 ± 0.38	2.66 ± 0.28	3.28 ± 0.43	0.288
*cd4-1*	1.57 ± 0.15	1.81 ± 0.25	1.50 ± 0.17	2.16 ± 0.29	0.237
*cd8a*	1.71 ± 0.19	1.69 ± 0.05	2.25 ± 0.29	2.05 ± 0.44	0.692
*cd8b*	0.40 ± 0.05	0.40 ± 0.04	0.49 ± 0.08	0.47 ± 0.17	0.669
*zap70*	2.41 ± 0.20	2.84 ± 0.23	2.65 ± 0.29	2.86 ± 0.29	0.573
*csf1r1*	0.84 ± 0.10	0.99 ± 0.07	1.18 ± 0.16	0.89 ± 0.13	0.176
*mrc1*	9.53 ± 0.60	8.77 ± 0.62	9.35 ± 0.69	9.39 ± 0.76	0.852
*tlr2*	4.15 ± 0.31	4.51 ± 0.41	3.85 ± 0.52	4.33 ± 0.40	0.702
*tlr5*	0.53 ± 0.03	0.46 ± 0.03	0.45 ± 0.04	0.39 ± 0.04	0.072
*tlr9*	0.85 ± 0.12	0.83 ± 0.13	0.56 ± 0.07	0.74 ± 0.08	0.194
*clec10a*	3.12 ± 0.99	1.78 ± 0.67	2.81 ± 0.66	1.49 ± 0.69	0.184
*igm*	361.86 ± 62.20	280.88 ± 49.03	354.97 ± 52.65	321.19 ± 77.33	0.762
*igtm*	13.86 ± 1.63^b^	13.91 ± 1.51^b^	16.40 ± 1.76^ab^	20.73 ± 2.50^a^	**0.049**
*b2m*	193.61 ± 28.95	257.31 ± 32.01	186.88 ± 17.39	198.69 ± 29.02	0.188
*casp3*	1.18 ± 0.05^b^	1.57 ± 0.17^a^	1.21 ± 0.05^ab^	1.18 ± 0.05^b^	**0.024**

*Note:* Values are the mean ± SEM of nine fish per experimental condition. All data are in reference to the expression values of *ck8/ccl20* of CTRL fish with an arbitrarily assigned value of 1. Different superscript lowercase letters indicate statistically significant differences among groups (one‐way ANOVA followed by Holm–Sidak post hoc test, *p* < 0.05). Significance of bold values was established in *p* < 0.05.

**Figure 1:**
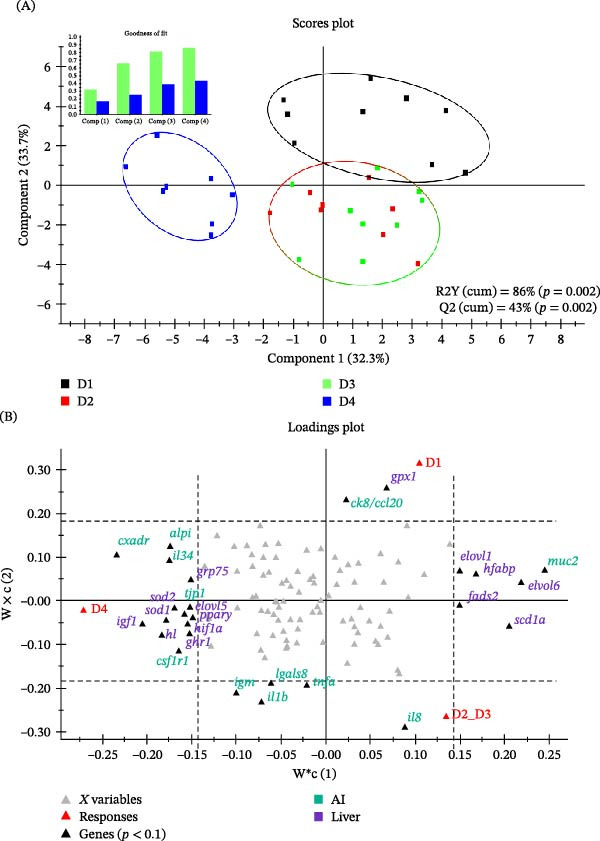
(A) Two‐dimensional PLS‐DA score plot of multi‐target (liver, anterior intestine and head kidney) gene expression, illustrating the distribution of the samples between the first two components in the model. The top‐left section of the figure displays the goodness of fit, representing the cumulative explained (R2Y(cum), green bars) and predicted (Q2(cum), blue bars) variance of each component. Values for these variances, along with *p*‐values from the permutation test, are located at the bottom‐right side of the figure. (B) Loading plot showing the genes that contribute most to group separation. *X*‐axis depicts the separation of experimental groups based on DHA‐rich microalgae oil inclusion; *Y*‐axis represents the separation between groups based on fish meal inclusion. Red triangles represent the experimental conditions used to represent multi‐tissular PLS‐DA (Figure 3A); grey triangles indicate genes, whereas black triangles indicate genes differentially expressed between the experimental groups. Gene names are coloured according to the assigned colour for each corresponding tissue.

According to the ANOVA results, multivariate analysis of gene expression (considering separately each tissue) failed to drive any group separation in the liver (Supporting Information 5: Figure S1A). By contrast, in both AI (Supporting Information 5: Figure S1B) and HK (Supporting Information 5: Figure S1C), the multivariate analysis was able to discriminate significantly (*p* = 0.002) two main groups corresponding to D1_D2_D3 and D4 fish, explaining the resulting PLS‐DA models more than 81%–90% of the observed variance (R2Y(cum)), and the 61%–69% of predicted variance (Q2(cum)). Such group differentiation was largely improved using a multi‐tissue approach, where the resulting PLS‐DA model clearly differentiated (R2Y(cum) = 86%; Q2(cum) = 43%; *p* = 0.002) up to three groups corresponding to D1, D2_D3 and D4 fish (Figure 1A). As a result of this, the loading plot analysis disclosed up to 11 and 15 discriminant genes in the AI and liver, respectively (Figure 1B). Specifically, along the axis‐Y, the up‐regulation of the hepatic glutathione peroxidase 1 (*gpx1*) and intestinal *ck8/ccl20* played a main role to distinguish D1 fish from the other three experimental groups (fishmeal‐free fed fish), while the up‐regulation of *il8* in the AI of D2_D3 fish mostly contributed to differentiate this group of fish from the other two extreme fish groups (D1 and D4). In contrast, the down‐regulation of four immune related genes (Immunoglobulin M, *igm*; *il1b*; galectin 8, *lgals8*; tumour necrosis factor α, *tnfα*) in the diets without fishmeal contributed equally to the group separation between D1, D2_D3 and D4 fish. Additionally, the separation along the X‐axis was driven by a set of genes that were specially up‐ (left side) or down‐regulated (right site) in the liver or AI of D4 fish, with a role in the regulation of somatic growth (GH receptor type 1, *ghr1*; *igf1*), intestinal barrier (*tjp1*; *alpi*; *cxadr*), immune function (*il34*; *csf1r1*), oxidative stress (superoxide dismutase [Cu–Zn], *sod1*; superoxide dismutase [Mn], *sod2*; glucose regulated protein 75 kDa, *grp75*; *hif1*α), lipid metabolism (*elovl1*; elongation of very long chain fatty acids 5, *elovl5*; *elovl6*; *scd1a*; *fads2*; *hl*; peroxisome proliferator‐activated receptor γ, *pparγ*; *hfabp*), and mucus production (*muc2*).

**Figure 2:**
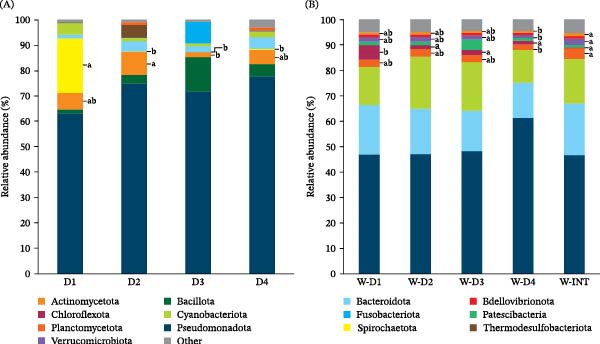
Relative abundance of bacterial phyla in (A) AI samples from fish fed D1, D2, D3 and D4, and (B) water samples from inlet (W‐INT) or tanks‐containing fish (W‐D1, W‐D2, W‐D3 and W‐D4). Significant differences (Kruskal–Wallis test, Dunn’s post‐test, *p* < 0.05) are indicated by different lowercase letters which correspond to pairwise comparisons within each phylum among groups.

**Figure 3:**
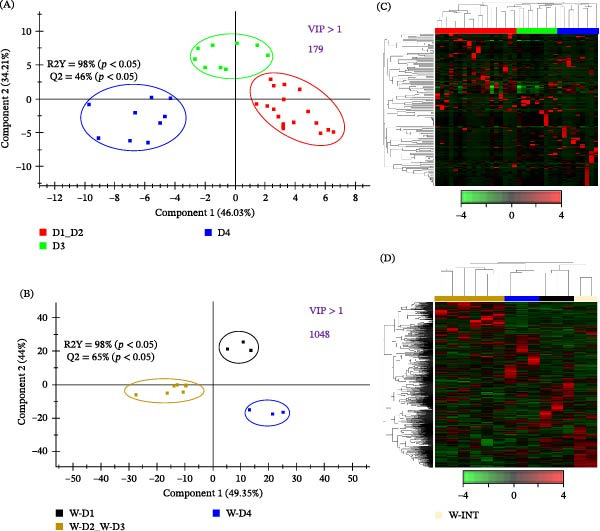
Two‐dimensional PLS‐DA scores plot representing the distribution of the dietary groups between the first two components in the model of (A) AI (D1, D2, D3 and D4) and (B) water from fish tanks (W‐D1, W‐D2, W‐D3 and W‐D4). The validation by the permutation test can be found in Supporting Information 6: Figure S2. Heatmaps representing the abundance distribution (*Z*‐score) of the taxa identified to be driving the separation by diet among all groups (VIP ≥1) for (C) AI and (D) water samples including W‐INT.

### 3.4. Microbial Richness, Alpha Diversity and Phylum Composition

Nanopore sequencing from the fish and water samples yielded 6,382,827 high quality reads, with a mean of 122,746 reads per sample. In total, 998 taxa were assigned to AI, and 3483 to water replicates from tanks containing fish, with rarefaction curves approximating saturation (Supporting Information 6: Figure S2). No significant changes in Chao1 and ACE richness indices, or Simpson and Shannon diversity indices, were found among experimental groups in any of the two types of samples (Supporting Information 7: Figure S3). Within each set of samples, average values of richness and diversity indices ranged between 400 and 600 (Chao1 and ACE), 1–1.5 (Shannon) and 0.5–0.8 (Simpson) in the AI, increasing in W‐D samples to 2400–2600, 4.5–4.75 and approximately 1, respectively. It is noteworthy that the trend for microbial richness and diversity in W‐INT samples was reduced in comparison to water samples from tanks containing fish (Supporting Information 7: Figure S3B).

At the phylum level (Figure 2), Pseudomonadota (formerly Proteobacteria) were the most abundant bacteria phylum, constituting more than 40%–60% of the total microbiota in all groups of AI and water samples. In parallel, a significant increase in the phylum Actinomycetota (formerly Actinobacteria) in fish fed D2 (~10%), compared with those receiving D3, was concurrent with an increase of Spirochaetota in animals fed D1 (~20%) in comparison to the rest of dietary groups (Figure 2A). In water samples, Bacteroidota (~20%) and Cyanobacteriota (~15%) were the second and the third most dominant phyla, but no significant changes were found among dietary groups or with respect to the W‐INT samples (Figure 2B). W‐INT group, however, showed an increased proportion of minor phyla such as Planctomycetota, Actinomycetota, and Verrucomicrobiota (vs. W‐D4), and Thermodesulfobacteriota (vs. W‐D1).

### 3.5. Microbiota Discriminant Analyses

**Figure 4:**
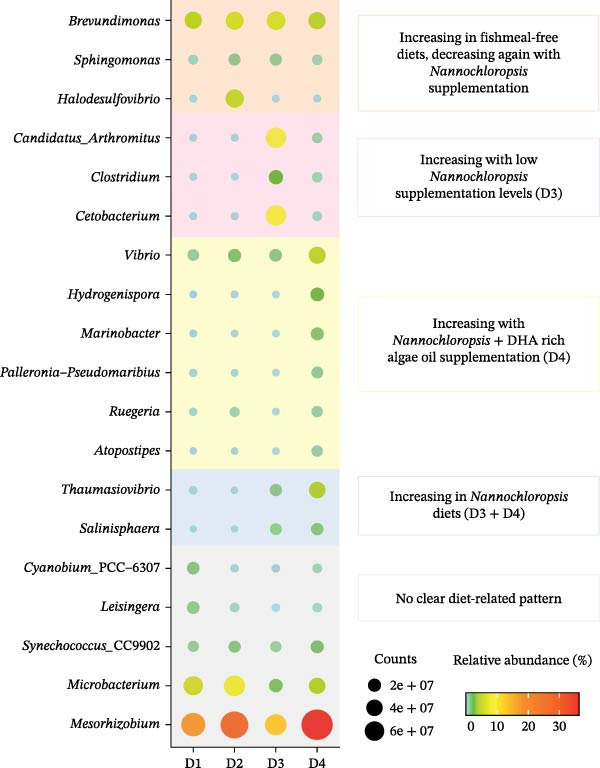
Dot plot map depicting taxa with more than 0.5% of proportion in at least one dietary group in AI samples from fish fed D1, D2, D3 and D4. The size of the dots represents the normalised counts in each group. The colour scale represents the mean abundance, in percentage, of each taxa within each group. Background colours indicate different patterns in abundance variation between groups.

Permutational multivariate analysis of variance test highlighted a statistically significant effect of dietary intervention upon the bacterial composition of fish AI (*p* = 0.039, *F* = 1.458, *R* ^2^ = 0.121) and water samples (*p* = 0.028, *F* = 1.681, *R* ^2^ = 0.427). To study in depth such effects, two separate PLS‐DA models were constructed with AI and water samples. In the case of AI, a fish from the D2 group was identified as outlier and discarded from the model. The remaining data were used to construct the final PLS‐DA model (R2Y = 98%, Q2 = 46%; Figure 3A), which clustered together D1 and D2 fish groups with a clear separation of fish fed D3 along the *Y*‐axis (34.21% of combined explained variance) and D4 in the *X*‐axis (46.03%). No outliers were detected in the model of water from tanks containing fish (R2Y = 98%, Q2 = 65%; Figure 3B), and the resulting PLS‐DA model clustered together W‐D2 and W‐D3 samples while separated W‐D1 and W‐D4 by both *X*‐ and *Y*‐axis (>93%). Both PLS‐DA models were successfully validated with a permutation test, discarding the possibility of over‐fitting of the supervised model (Supporting Information 8: Figure S4). The heatmap representation (Figure 3C,D) of bacterial taxa with a VIP ≥1 drove the correct ordination of all individuals in each group with 179 discriminant bacterial taxa in AI and 1045 in water samples from tanks with/without fish. The taxa overcoming the above clustering ordination were used for an additional filtering approach based on a minimum abundance of 0.5%. After the application of this filter, a total of 20 and 27 taxa were selected from AI and water samples, respectively. In the case of AI (Figure 4), the genus *Mesorhizobium* was the most abundant taxa in all dietary groups, with a 16%. relative abundance in D1 that increased up to 25% in D2 fish, decreased up to 12% in D3 fish, but increased again (36%) in D4 fish with the highest dietary inclusion level of microalgae ingredients. Therefore, the observed abundance changes of this highly represented taxa did not appear to follow a clear dietary pattern, as it was also the case for other bacterial taxa (*Cyanobium*_PCC‐6307, *Leisingera*, *Synechococcus*_CC9902 and *Microbacterium*). Conversely, the abundances of *Brevundimonas*, *Sphingomonas* and *Halodesulfovibrio* increased in fish fed the fishmeal‐free D2, and returned to levels found in the control diet (D1) with the inclusion of *Nannochloropsis* and DHA‐enriched microalgae oil. Moreover, the greater abundance of three bacterial genera (*Candidatus*_*Arthromitus*, *Clostridium* and *Cetobacterium*) was specifically associated to D3 fish. Likewise, a higher abundance of other six bacterial genera (*Vibrio*, *Hydrogenispora*, *Marinobacter*, *Palleronia*‐*Pseudomaribius*, *Ruegeria* and *Atopostipes*) appeared in D4 fish, whereas the abundance of *Thaumasiovibrio* and *Salinisphaera* genera was increased in both D3 and D4 fish.

**Figure 5:**
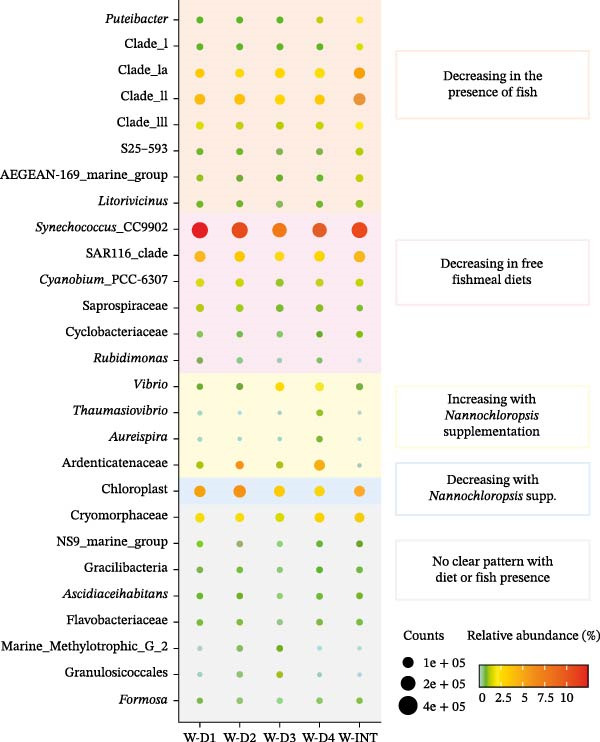
Dot plot map depicting taxa with more than 0.5% of proportion in at least one group in water samples from inlet (W‐INT) or tanks‐containing fish (W‐D1, W‐D2, W‐D3 and W‐D4). The size of the dots represents the normalised counts in each group. The colour scale represents the mean abundance, in percentage, of each taxa within each group. Background colours indicate different patterns in abundance variation between groups.

In water samples (Figure 5), the genus *Synechococcus*_CC9902 was the most abundant (8%–12%) across all experimental samples (including W‐INT samples, 10.8%), though its abundance decreased in water samples from tanks fed fishmeal‐free diets (W‐D2, W‐D3 and W‐D4). Five additional bacterial taxa, including the genera *Cyanobium*_PCC‐6307 and *Rubidimonas*, the families Saprospiraceae and Cyclobacteriaceae and the SAR116 clade, exhibited a similar pattern. The abundance of other five bacterial taxa varied specifically with *Nannochloropsis* supplementation. Four of these taxa increased in abundance (*Vibrio*, *Thaumasiovibrio* and *Aureispira* genera, and Ardenticatenaceae family), while one decreased (undefined Cyanobacteria and Chloroplast). Conversely, in comparison to W‐INT samples, eight bacterial taxa decreased its abundance with the presence of fish in the tank regardless of the diet. Such set of bacteria included four taxa from the *Pelagibacterales* order (Clades I, Ia, II and III), the genera *Puteibacter* and *Litorivicinus*, and the taxa associated to the S25−593 and AEGEAN‐169_marine_group genera. Lastly, as also reported in the AI, the abundance of some bacterial taxa (*Cyanobium*, *Leisingera*, *Synechococcus*, *Microbacterium* and *Mesorhizobium*) did not appear to be linked to a given diet or to the presence of fish in the tank.

## 4. Discussion

The use of microalgae in aquaculture has evolved from traditional live feed for larvae to practical dietary supplements and feed replacements in both juvenile and adult stages. However, an integrative approach covering the whole response of the organism and its microbiota is still lacking to disclose the combined mechanisms exerted by the fish under microalgae nutritional intervention. The present study evaluated the systemic, molecular and microbial responses of gilthead sea bream to innovative, fishmeal‐free diets enriched with *Nannochloropsis* biomass and DHA‐rich algae oil. The integration of transcriptomic and microbial data demonstrates that both feed ingredients significantly influenced key performance parameters and physiological adaptive features, thereby supporting the use of circular feed ingredients in sustainable aquaculture. At the end of the trial, fish fed fishmeal‐free diets (D2–D4) showed growth performance largely comparable to that of the control diet group (D1), with only a slight reduction in final body weight. This apparent growth deceleration was likely accentuated by the low variability among tank replicates, which amplified minimal differences and rendered them statistically significant. Alternatively, these differences may reflect a nutritional and physiological adjustment associated with the transition from a commercial fishmeal‐based diet to fishmeal‐free formulations, a process that may have been favoured in the control diet group (D1). The inexistence of growth reduction in D2–D4 diets did not make us to make digestibility assays with yttrium. Thus, we could not determine whether differences in digestible protein or digestible energy and its relation with growth. What is indeed clear is, certainly, that at the end of trial, overnight fasted D4 fish showed the highest hepatic *igf1* expression, the main mediator of GH’s systemic growth‐promoting effects, which are triggered when GH binds to hepatic GH receptors [59]. Thus, very often the hepatic expression of *igf1* and *ghr1* are strongly correlated, serving both genes to establish the discriminant transcriptional feature of D4 fish in our multi‐variate and multi‐tissue PLS‐DA approach. This observation is, at least at the transcriptional level, consistent with modulation of the GH/IGF axis, which may in turn support an improved growth‐related transcriptional profile in D4 fish. However, the experimental design does not allow us to determine whether this effect is attributable to the increased supply of *Nannochloropsis* or to the higher inclusion of DHA‐rich microalgae oil. Additionally, from a mechanistic perspective, future studies would benefit from investigating the specific effects of modifying EPA:DHA and n‐3:n‐6 ratios in microalgae‐ and DHA‐rich microalgae oil enriched diets, which opens the door to study the influence of these ingredients on membrane composition and eicosanoid production. In any case, the present study unlocks the potential of *Nannochloropsis* for the valorisation of brewery waste into a sustainable fishmeal‐free diet for a marine carnivorous fish such as gilthead sea bream, in which plant protein ingredients were partially replaced by microalgae protein extracts as a more sustainable dietary protein source. Moreover, future research would benefit from incorporating temporal transcriptomic analyses in long‐term trials to better link dietary adaptation with time‐dependent transcriptional responses.

Circulating levels of cholesterol were lowered in D3 fish, while TG were increased in both fish fed D3 and D4 diets. In the case of fish fed the D4 diet, current histopathological studies indicate that this effect coincided with a low abundance of hepatocyte/enterocyte vacuolisation in the post‐absorptive state [59], which was likely reinforced by the DHA surplus in the D4 diet [60]. All this together is indicative of a different regulation of lipid digestion and metabolism with the addition of *Nannochloropsis* and DHA‐enriched microalgae oil in D3 and D4 diets. This assumption was reinforced by a pronounced anti‐lipogenic effect in D4 fish that rendered a reduced liver weight and HSI, in concurrence with a marked depletion of hepatocyte lipid depots that was evidenced by the histological survey of liver tissue sections [59]. After a 24 h fasting period in our experimental design, at the transcriptional level, this metabolic feature occurred in parallel with a down‐regulated expression of lipogenic enzymes (*elovl1*, *elovl6*, *fads2* and *scd1a*) and intracellular long‐chain fatty acid fatty acids transporters (*hfabp*). Conversely, the hepatic expression of *elovl5*, required for the inhibition of lipogenic enzymes [61], was up‐regulated in D4 fish, reinforcing the anti‐lipogenic profile of this group of fish. At the same time, the hepatic expression of *hl*, a lipolytic enzyme that hydrolyses TG and phospholipids present in circulating plasma lipoproteins, was up‐regulated in D4 fish. All this together indicates a switch in lipid metabolism towards tissue fatty acid uptake rather than de novo fatty acid synthesis in the liver of 24 h‐fasted D4 fish. This feature would help maintain a controlled hypolipidemic tone with the increase of plasma TG levels in D4 fish. The ultimate mechanisms remain unclear, but the up‐regulation of *pparγ* and *hif1*α transcription factors would contribute to preserving the delicate balance of mechanisms regulating lipogenesis and oxidative stress pathways. Certainly, oxidative stress triggers lipogenic activity in primary cultures of hepatocytes by activating fatty acid synthesis [62], but the opposite is also true and herein the increase of plasma TAC was especially evident with the blockage of the hepatic lipogenic activity in D4 fish. Nonetheless, we acknowledge that the magnitude of the anti‑lipogenic response in D4 fish may indicate a narrower metabolic buffering and energy mobilisation capacity under prolonged fasting or additional stressors, and future studies should evaluate whether this remodelled lipid‑metabolic state remains advantageous across more demanding physiological contexts to fully understand the potential of *Nannochloropsis*‐based diets for aquaculture.

The links between a specific nutrient and the changing regulatory mechanisms of lipid metabolism remains elusive in our experimental model, though it is well known the dual role of n‐3 long‐chain PUFA as antioxidant and anti‐steatosis agents [60]. This was supported herein by the tissue specific up‐regulated expression of several antioxidant markers (*sod1*, *sod2* and *grp75*) in the liver of D4 fish. Otherwise, the second line of defence against reactive oxygen species (ROS) involves non‐enzymatic antioxidant mechanisms that are represented by endogenous and dietary antioxidants molecules, characterised by their ability to rapidly inactivate radicals and oxidants. Such compounds include among others vitamin E, vitamin C, carotenoids, some minerals (e.g., Zn, Mn, Cu, Se) and polyphenols (flavonoids, phenolic acids, stilbenes and lignans), which are especially abundant in micro/macro algae extracts. Thus, a number of studies have associated algae‐enriched diets with radical scavenging and singlet O_2_ quenching activity as part of the host defence mechanisms against extreme oxidative environmental conditions [35, 63–65].

The intestinal pro‐inflammatory action is a well‐known drawback effect of low fishmeal‐based diets in marine farmed fish, which can be partially or fully reversed through the action of feed additives, including among others feeding attractants, prebiotics, probiotics, phytobiotics, lipid emulsifiers and organic acids [66–69]. In this case, experimental evidence revealed the absence of clear histopathological signs of intestinal inflammation in any experimental group [59]. However, a number of pro‐inflammatory markers (*igm*, *lgals8*, *il1b* and *tnfa*) were up‐regulated in fish fed the fishmeal‐free diets (D2–D4) in comparison to the control diet fish (D1 fish). Additionally, another pro‐inflammatory marker, such as *il8* was specifically up‐regulated in D2 and D3 fish, but not in D4 fish. Likewise, in D4 fish, the up‐regulated expression of *il34*, a relatively newly cytokine with both pro‐inflammatory and anti‐inflammatory actions, was positively co‐regulated with the expression of its binding macrophage receptor (*csf1r1*) [70]. Binding of this interleukin activates several signalling pathways crucial for immune surveillance during the inflammatory response, promoting, through the dual role of *il34* role, a shift towards the processes that govern the inflammation resolution [71]. In the same way, *alpi*, a brush border digestive enzyme, considered an inherited cause of inflammatory bowel diseases in humans [72], was up‐regulated in D4 fish. Markers of epithelial barrier also showed a complex balance, exemplified by the reduced expression of *muc2*, a major component of intestinal mucus in fish and gilthead sea bream in particular [73], which was negatively co‐regulated with *cxadr*, a homophilic cell adhesion molecule that is essential for tight junction integrity [74]. Although histology results showed no histopathological damage (not appreciated differences in the abundance of intraepithelial lymphocytes, degree of submucosal hyperplasia or goblet cells) [59], further functional immune assays would be valuable to elucidate the biological relevance of the observed transcriptional responses in terms of host defence under pathogen exposure.

As a general trend, microbial richness and evenness in the water samples from tanks‐containing fish was 2–6 times higher than in AI, regardless of the experimental group. The diet had a significant impact on the composition of the adherent bacterial communities inhabiting the AI, with 179 bacteria taxa with a discriminant role among groups. Comparatively, the number of discriminant bacteria (1048) was largely increased in water samples due, at least in part, to a lower replicate variability that allows to detect minor changes in microbiota composition as a reliable variation. This reinforces the intestine’s enclosed nature, which must preserve its function as much as possible, minimising microbial changes compared to other compartments that are more exposed to the environment, such as the water, the skin or the gills [75]. Certainly, in our experimental setup, the observed changes in the water microbial composition occurred despite the weekly cleaning tasks and the daily total water renovation each 2.5–3 h, which reveals the importance of the presence of active feeding fish and the type of feeding regimen in influencing the microbiota of the water surrounding fish. Thus, some typical Mediterranean shore bacteria [76], such as *Puteibacter*, *Pelagibacterales*, *AEGEAN-169 marine group* and *Litorivicinus* taxa, were displaced by other bacterial groups in the presence of active feeding fish. Moreover, the abundance of some bacteria taxa (*Cyanobium*, Saprospiraceae, Cyclobacteriaceae and *Rubidimonas*) was specifically decreased in the presence of fish fed fishmeal‐free diets. Fishmeal is a protein‐rich product, and these taxonomic groups appear to play a key role in decomposing protein‐sized nitrogen compounds from this feed ingredient [77]. However, in the absence of fishmeal, these bacteria taxa would become unnecessary or replaceable by other microorganisms. Thus, up to four water bacteria taxa were increased in *Nannochloropsis*‐ and DHA‐rich algae oil related D3–D4 fed fish. Among them, the *Vibrio* and *Thaumasiovibrio* taxa also shared an increased abundance in the AI of D4 fish, which would show some connexion between the intestinal bacterial population and that of the fish surrounding water. Besides this, the other two bacteria taxa (*Aureispira*, Ardenticatenaceae) included in this category are able to degrade organic carbon sources contributing to the overall detoxification and recycling of organic matter, maintaining water quality as already stated in some studies undergone with water samples of the Black Sea [78, 79].

*Mesorhizobium* was the most abundant genus in the adherent AI microbiota of all the experimental groups, ranging from 12% to 16% in D1 and D3, to 25% in D2 and 36% in D4. Species of this genus are well‐known symbiotic organisms of soya and algae [80], and its presence in the gut microbiota of gilthead sea bream is becoming a common feature in several studies with plant‐based diets, though it is usually quantified as less than 1% of the total gut microbial population [48, 81]. These findings may suggest that some dietary raw materials represent a good matrix for the *Mesorhizobium* proliferation, but it is challenging to correlate changes in gut abundance with specific changes in nutrient composition, when comparing free fishmeal‐based diets (D2–D4) either among them or to the control diet (D1). Further studies using metagenomic or metatranscriptomic approaches would be required to determine whether this genus contributes actively to nutrient processing or represents an allochthonous, diet‐associated transient taxon. The same occurs with *Microbacterium*, a common microorganism inhabiting the intestine of farmed fish [69], but also with *Cyanobium*, *Leisingera* and *Synechococcus* genera that are more related with marine environments rather than with the microbial population inhabiting the intestine [76, 82].

In contrast to the earlier findings, there is also now evidence that the reshaping of gut microbiota is a permissive, but also necessary process for the long‐term preservation of growth performance and gut homeostasis to cope with changes in diet composition [83, 84]. Thus, we found herein a very sharply pattern of *Halodesulfovibrio* (5% in D2), which was nearly completely reversed by *Nannochloropsis*‐based diets, with and without DHA surplus. This feature has been considered beneficial because *Halodesulfovibrio* has the potential under anoxic conditions to biodegrade organosulfur compounds by its transformation into hydrogen sulphide (H_2_S), which has been linked in the human gut to the development of various diseases due to the corrosive and toxicity action of H_2_S on cellular membranes [85, 86]. Another beneficial action on gut microbiota was related to a higher abundance in D3 fish of *Arthromitus* and *Cetobacterium*. Certainly, *Arthromitus* stimulates the production of regulatory T cells in the gut‐associated lymphoid tissue (GALT), which is becoming crucial for suppressing excessive immune responses and maintaining immune tolerance [87]. Likewise, *Cetobacterium* is a good producer of short‐chain fatty acids (SCFAs) through the saccharolytic fermentation of carbohydrates that escape digestion and absorption in the intestinal tract [88]. The major products of this fermentation are acetate, propionate and butyrate with a number of local effects, ranging from the maintenance of intestinal motility and barrier integrity, mucus production and immune function in a wide range of organisms including fish [66, 89]. *Salinisphaera* is another bacterial genus associated with the production of SCFA [90], but in this case this bacterial taxon was overrepresented in both fish fed D3 and D4 diets, which are enriched in polysaccharides with the *Nannochloropsis* and DHA‐rich oil supplementation. A similar trend in response to dietary challenges was reported for *Thaumasiovibrio*, a bacterial genus that also becomes overrepresented in gilthead sea bream following the recovery phase of antibiotic exposure [91]. Otherwise, a number of bacterial taxa (*Hydrogenispora*, *Marinobacter*, *Palleronia*‐*Pseudomaribius*, *Ruegeria* and *Atopostipes*), all of them associated with a healthy core microbiota rather than to a pathogenic behaviour [92], were exclusively overrepresented in D4 fed fish shaping more than 10% of the total microbial population. This finding supports the notion that dietary composition not only shapes overall performance, but also promotes the proliferation of beneficial microbial taxa. Thus, *Hydrogenispora* may carry out fermentative action on carbohydrates due to the sugar‐rich composition of the microalgae [93, 94]. *Palleronia*–*Pseudomaribius* has been linked to environmental stability through the formation of stable biofilms in larval coral microbiomes dominated by algae [95]. *Marinobacter* participates in detoxifying harmful substances, and polysaccharide extracts from this genus have recently been identified as neuroprotective in Nile tilapia [96]. Similarly, *Ruegeria* appears capable of producing antimicrobial compounds that inhibit the growth of pathogenic bacteria [97], as demonstrated recently through in vitro assays that assessed the anti‐pathogen efficacy of both individual bacterial cultures and mixed algal microbiomes [98]. Finally, *Atopostipes* belongs to the lactic acid bacteria family, which very often produces by‐products with bioactivity and a diverse range of health‐promoting effects, including protection against infectious agents, immunomodulatory effects, anti‐allergenic effects, anti‐obesity effects, anti‐oxidant effects, enhancing the bioavailability of vitamins/minerals, anti‐anxiety effects in humans and a wide range of animals including fish [99]. Overall, although the temporal dynamics and mechanistic links between algal polysaccharides, host physiology, and microbiota remain unclear within the present experimental design, the final microbiota snapshots suggest that the microbial community is a relevant target in studies using *Nannochloropsis*‐based diets. However, the specific dietary components responsible of the observed effects remain largely unexplored, and further research should investigate the role of algal bioactive compounds (e.g., carotenoids, polyphenols and vitamins) as well as plant‐derived antinutritional factors (e.g., phytates, saponins and tannins) in driving the observed transcriptional and microbiota alterations.

## 5. Conclusion

*Nannochloropsis oceanica* harvested from brewery wastewater cultivation was included in gilthead sea bream diets and found to be able to provide beneficial effects on growth performance and antioxidant capacity, probably as a result of the combined action of enzymatic and non‐enzymatic antioxidant mechanisms. Multi‐tissue gene expression approaches revealed marked changes in lipid metabolism at the highest inclusion level of *Nannochloropsis*. In D4 fish, coinciding with a DHA surplus, this shift favoured tissue fatty acid uptake and transport while blocking hepatic lipogenesis. In parallel, the gut microbiota of *Nannochloropsis*‐fed fish showed enhanced detoxifying, anti‐oxidant and anti‐inflammatory properties, which would allow to alert the host immune system without triggering maladaptive inflammation at the tissue level. At the end, this is the result of a delicate equilibrium between the gut microbiota and the host transcriptomics, which serves to depict the most favourable condition to prevent the attack of invading pathogens maintaining under control the response of the host immune system. Altogether, this integrative approach remarks the importance of a multi‐layer point of view when analysing the effects of a dietary intervention based on the valorisation of waste products, and the use of new fishmeal‐free feed formulations for farmed fish.

## Author Contributions

**Fernando Naya-Català:** data curation, formal analysis, investigation, software, visualisation, supervision, writing – original draft, writing – review and editing. **Ricardo Domingo-Bretón** **, Paul George Holhorea and Federico Moroni**: data curation, formal analysis, investigation, software, visualisation, writing – review and editing. **Qinge Ma, Belenguer Álvaro and Josep Calduch-Giner:** investigation, writing – review and editing. **Alla Silkina:** conceptualisation, project administration. **Danny Van Mullem and Ronald Halim:** conceptualisation, funding acquisition, project administration, resources, writing – review and editing. **Jaume Pérez-Sánchez:** conceptualisation, data curation, formal analysis, funding acquisition, investigation, methodology, project administration, resources, supervision, visualisation, writing – original draft, writing – review and editing.

## Funding

This work was funded by the ‘AlgaeBrew: Unlocking the potential of microalgae for the valorisation of brewery waste products into omega‐3 rich animal feed and fertilisers’ transnational EU project (ERA‐NET’s SUSFOOD2‐FOSC joint call in innovative solutions for resilient, climate‐smart and sustainable food systems; Project ID 2021SUSFOODFOSCEN101). The authors would like to acknowledge the Department of Agriculture, Food and the Marine (DAFM), Ireland, for their financial support. Fernando Naya‐Català was funded by the CSIC‐MOMENTUM (DigiAcuaSOS; MMT24‐IATS‐01‐01) postdoctoral contract, funded by the MRR–Next Generation EU, within the framework of the General Call of the public business entity Red.es to participate in talent attraction and retention programs under Investment 4 of Component 19 of the Recovery, Transformation and Resilience Plan (PRTR). Federico Moroni was funded by the Generalitat Valenciana through the postdoctoral programme APOSTD (Grant CIAPOS/2024/092), co‐funded by the European Union through the European Social Fund Plus (FSE+).

## Conflicts of Interest

Danny Van Mullem reports a relationship with LSAQUA that includes: employment. The other authors declare no conflicts of interest.

## Supporting Information

Additional supporting information can be found online in the Supporting Information section.

## Supporting information


**Supporting Information 1** Table S1. Ingredients and chemical composition of experimental diets.

**Supporting Information 2** Table S2. Fatty acid composition of experimental diets (% total FA methyl esters).

**Supporting Information 3** Table S3. Primers for qPCR amplification of hepatic transcripts.

**Supporting Information 4** Table S4. Primers for qPCR amplification of anterior intestine ( ^∗^) and head kidney (†) transcripts.

**Supporting Information 5** Figure S1. Two‐dimensional PLS‐DA score plots of (A) liver, (B) anterior intestine and (C) head kidney gene expression, representing the distribution of the samples between the first two components in the model. The cumulative explained R2Y(cum) and predicted Q2(cum) variance, as well as the *p*‐values of the permutation plot can be found at the bottom‐right side of the figure.

**Supporting Information 6** Figure S2. Permutation plots of the PLS‐DA model represented in Figures 1 and 3.

**Supporting Information 7** Figure S3. Rarefaction curves obtained from the sequencing data of the 50 samples included in this study.

**Supporting Information 8** Figure S4. Species richness estimators (Chao1 and ACE) and diversity indexes (Shannon and Simpson) of (A) AI samples from fish fed D1, D2, D3 and D4, and (B) water samples from inlet pipe (W‐INT) or tanks‐containing fish (W‐D1, W‐D2, W‐D3 and W‐D4).

## Data Availability

Raw sequencing data are available at NCBI’s Sequence Read Archive under accession PRJNA1335540 (BioSample accession numbers: SAMN51991205‐254).
