# Supplementary material for: From Brewery Waste to Sustainable Aquafeed: Harnessing Nannochloropsis Microalgae for Fishmeal‐Free Gilthead Sea Bream Diets
Source: Aquac Nutr. 2026 May 21;2026:1003936. doi: 10.1155/anu/1003936 (PMC13191778; doi:10.1155/anu/1003936)
Supplement: Supplementary file 1 — Supporting Information 1 Table S1. Ingredients and chemical composition of experimental diets. [file ANU-2026-1003936-s007.docx]

**Supplementary Table 1**. Ingredients and chemical composition of experimental diets.

| Ingredient (%) |  |  | | |  |
| --- | --- | --- | --- | --- | --- |
|  | D1 | D2 | D3 | D4 | |
| Fishmeal 60 | 15 |  |  |  | |
| *Nannochloropsis* meal |  |  | 2 | 14 | |
| Soybean meal | 12.7 | 25.8 | 17.8 | 8.4 | |
| Wheat meal | 18.8 | 14.4 | 14.8 | 11 | |
| Wheat gluten | 10 | 10 | 10 | 10 | |
| Rapeseed meal | 10 | 8 | 10 | 7 | |
| Poultry meal^1^ | 18.3 | 21.9 | 25 | 25 | |
| Feathermeal hydrolysate^2^ |  |  |  | 3 | |
| Porcine blood meal^3^ | 2 | 2.2 | 2 | 2 | |
| Single cell protein U |  | 5 | 5 | 5 | |
| Soya oil | 7.36 | 4.94 | 5.50 | 4.27 | |
| Fish oil | 3.89 | 4.81 | 4.57 |  | |
| DHA-rich microalgae oil (*Schizochytrium*) |  |  |  | 7.6 | |
| L-Lysine HCl | 0.037 | 0.267 | 0.309 | 0.513 | |
| DL-Methionine |  | 0.07 | 0.054 | 0.064 | |
| Vitamin and mineral premix^4^ | 0.5 | 0.5 | 0.5 | 0.5 | |
| C-Force AQF^5^ | 0.4 | 0.4 | 0.4 | 0.4 | |
| Monocalcium phosphate | 0.40 | 1.12 | 0.82 | 1.01 | |
| Calcium carbonate | 0.50 | 0.45 | 1.07 | 0.02 | |
| Lysoforte emulsifier | 0.05 | 0.05 | 0.05 | 0.05 | |
|  |  |  |  |  | |
| *Proximate* *composition* |  |  |  |  | |
| Dry matter (%) | 94.9 | 95.2 | 95.7 | 93.7 | |
| Crude protein (% DM) | 42 | 42 | 42 | 42 | |
| Crude fat (% DM) | 18 | 18 | 18 | 18 | |
| Crude Ash (% DM) | 6.15 | 7.99 | 8.82 | 10.51 | |

^1^Poultry meal, Sonac 70; ^2^Premium hydrolysed protein meal produced from selected and purified chicken feathers, SONAC; ^3^Hemoglobin 92P, Sonac; ^4^The inclusion of 0.5% of the vitamin and mineral premix contributed to an additional supply of the following micronutrients: Vitamins (IU or mg/kg diet): vitamin A, 8,000 IU; vitamin D_3_, 2,500 IU; vitamin C, 205 mg; vitamin E, 200 mg; vitamin K_3_, 8 mg; vitamin B_1_, 15 mg; vitamin B_2_, 20 mg; pantothenic acid, 40 mg; vitamin B_6_, 20 mg; vitamin B_12_, 0.05 mg; niacin, 120 mg; folic acid, 7 mg; biotin, 1 mg; betaine, 240 mg. Minerals and trace elements (mg or %/kg diet): iron, 50 mg; copper, 5 mg; manganese, 15 mg; zinc, 70 mg; iodium, 1.25 mg; selenium, 0.3 mg; calcium, 0.05%; digestible phosphorus, 0.12% (VDS Fish 0,5%, VDS, Deerlijk, Belgium); ^5^Chemical composition (% on dry matter basis): crude protein 2.56%; fat, 11.42%; crude fiber, 0.64%, ash 55.93%, calcium 12.11%, phosphorus 0.19%, sodium 0.05% (VDS, Deerlijk, Belgium).
