# Supplementary material for: From Brewery Waste to Sustainable Aquafeed: Harnessing Nannochloropsis Microalgae for Fishmeal‐Free Gilthead Sea Bream Diets
Source: Aquac Nutr. 2026 May 21;2026:1003936. doi: 10.1155/anu/1003936 (PMC13191778; doi:10.1155/anu/1003936)
Supplement: Supplementary file 2 — Supporting Information 2 Table S2. Fatty acid composition of experimental diets (% total FA methyl esters). [file ANU-2026-1003936-s001.docx]

| **Supplementary Table 2**: Fatty acid composition of experimental diets (% total FA methyl esters). | | | | |
| --- | --- | --- | --- | --- |
| Fatty acid (%) | **D1** | **D2** | **D3** | **D4** |
| 14:0 | 1.65 | 1.74 | 1.70 | 2.47 |
| 15:0 | 0.12 | 0.11 | 0.11 | 0.13 |
| 16:0 | 14.80 | 15.09 | 15.48 | 19.52 |
| 16:1n-9 | tr | 0.21 | 0.22 | tr |
| 16:1n-7 | 2.45 | 2.60 | 3.21 | 5.83 |
| 16:2 | 0.08 | 0.05 | tr | tr |
| 17:0 | 0.17 | 0.16 | 0.15 | 0.13 |
| 16:3 | 0.11 | 0.08 | 0.06 | 0.14 |
| 17:1 | 0.11 | 0.06 | 0.06 | tr |
| 18:0 | 4.60 | 4.28 | 4.30 | 3.56 |
| 18:1n-9 | 23.14 | 23.26 | 23.12 | 18.27 |
| 18:1n-7 | 1.62 | 2.93 | 3.51 | 2.52 |
| 18:1n-5 | 0.05 | 0.20 | 0.15 | 0.12 |
| 18:2n-6 | 36.92 | 35.20 | 35.12 | 29.19 |
| 18:3n-3 | 4.66 | 4.85 | 4.62 | 3.26 |
| 18:4n-3 | 0.41 | 0.44 | 0.36 | 0.08 |
| 20:0 | 0.30 | 0.24 | 0.22 | 0.21 |
| 20:1n-11 | 0.09 | 0.09 | 0.07 | tr |
| 20:1n-9 | 1.14 | 1.30 | 1.12 | 0.17 |
| 20:1n-7 | 0.06 | 0.08 | 0.07 | tr |
| 20:2n-6 | 0.16 | 0.17 | 0.16 | 0.06 |
| 20:3n-6 | 0.07 | 0.07 | 0.08 | 0.16 |
| 20:4n-6 | 0.42 | 0.42 | 0.55 | 1.30 |
| 20:3n-3 | 0.05 | 0.05 | 0.04 | tr |
| 20:4n-3 | 0.18 | 0.19 | 0.16 | 0.13 |
| 20:5n-3 (EPA) ^1^ | 2.26 | 2.06 | 2.06 | 2.52 |
| 22:0 | 0.22 | 0.15 | 0.14 | 0.16 |
| 22:1n-11 | 1.00 | 1.13 | 0.87 | tr |
| 22:1n-9 | 0.15 | 0.16 | 0.13 | tr |
| 22:4n-6 | 0.06 | 0.12 | 0.12 | 0.12 |
| 22:5n-6 | 0.08 | 0.07 | 0.06 | 2.45 |
| 22:5n-3 | 0.40 | 0.43 | 0.33 | 0.06 |
| 24:0 | 0.06 | tr | tr | tr |
| 22:6n-3 (DHA) ^2^ | 2.34 | 1.96 | 1.67 | 7.48 |
| 24:1 | 0.10 | 0.06 | tr | tr |
|  |  |  |  |  |
| Ʃ SFAs ^3^ | 21.91 | 21.76 | 22.09 | 26.16 |
| Ʃ MUFAs ^4^ | 29.90 | 32.08 | 32.52 | 26.91 |
| Ʃ n-6 PUFAs ^5^ | 37.71 | 36.04 | 36.08 | 33.27 |
| Ʃ n-3 PUFAs ^6^ | 10.29 | 9.99 | 9.24 | 13.52 |
| n-3/n-6 PUFAs^5,6^ | 0.27 | 0.27 | 0.25 | 0.41 |
| EPA + DHA | 4.60 | 4.03 | 3.73 | 10.00 |
| EPA/DHA | 0.96 | 1.05 | 1.24 | 0.34 |

^1^ EPA, eicosapentaenoic acid

^2^ DHA, docosahexaenoic acid

^3^ Ʃ SFAs, Ʃ saturated fatty acids

^4^ Ʃ MUFAs, Ʃ monounsatured fatty acids

^5^ Ʃ n-6 PUFAs, Ʃ n-6 Polyunsaturated fatty acids

^6^ Ʃ n-3 PUFAs, Ʃ n-3 Polyunsaturated fatty acids
