# Supplementary material for: From Brewery Waste to Sustainable Aquafeed: Harnessing Nannochloropsis Microalgae for Fishmeal‐Free Gilthead Sea Bream Diets
Source: Aquac Nutr. 2026 May 21;2026:1003936. doi: 10.1155/anu/1003936 (PMC13191778; doi:10.1155/anu/1003936)
Supplement: Supplementary file 3 — Supporting Information 3 Table S3. Primers for qPCR amplification of hepatic transcripts. [file ANU-2026-1003936-s002.docx]

**Supplementary Table 3**. Primers for qPCR amplification of hepatic transcripts.

| **Gene** | **Symbol** | **GenBank** | **Primer** |
| --- | --- | --- | --- |
| Beta-actin | *actb* | KY388508 | F: TCC TGC GGA ATC CAT GAG A |
|  |  |  | R: GAC GTC GCA CTT CAT GAT GCT |
|  |  |  |  |
| Adipose triglyceride lipase | *atgl* | JX975711 | F: GTG CTT CAG TCC TGG ATG TCT TC |
|  |  |  | R: AGC CTT GCA GGT CCA TGT TGA |
|  |  |  |  |
| Cytochrome c oxidase subunit 1 | *cox1* | KC217652 | F: GTC CTA CTT CTT CTG TCC CTT CCT GTT CT |
|  |  |  | R: AGG TTT CGG TCT GTA AGG AGC ATT GTA ATC |
|  |  |  |  |
| Cytochrome c oxidase subunit 2 | *cox2* | KC217653 | F: ACT GCC TAC ACA GGA CCT TGC C |
|  |  |  | R: GTC TGC TTC CAG GAG ACG GAA TTG T |
|  |  |  |  |
| Carnitine palmitoyltransferase 1a | *cpt1a* | JQ308822 | F: GTG CCT TCG TTC GTT CCA TGA TC |
|  |  |  | R: TGA TGC TTA TCT GCT GCC TGT TTG |
|  |  |  |  |
| Citrate synthase | *cs* | JX975229 | F: TCC AGG AGG TGA CGA GCC |
|  |  |  | R: GTG ACC AGC AGC CAG AAG AG |
|  |  |  |  |
| Superoxide dismutase [Cu-Zn] | *cu-zn-sod / sod1* | JQ308832 | F: TCA CGG ACA AGA TGC TCA CTC TC |
|  |  |  | R: GGT TCT GCC AAT GAT GGA CAA GG |
|  |  |  |  |
| Cholesterol 7-alpha-monooxygenase | *cyp7a1* | KX122017 | F: CCC TGC TAT TAA AGT CCC ACC TCT |
|  |  |  | R: ATC GTA GGT AGG CTG GAG GAT TC |
|  |  |  |  |
| Elongation of very long chain fatty acids 1 | *elovl1* | JX975700 | F: CTT CCT ACA CAT CTT CCA CCA CTC |
|  |  |  | R: CCA TTC CAC CAG GAG CAA AGG |
|  |  |  |  |
| Elongation of very long chain fatty acids 4 | *elovl4* | JX975701 | F: CGG TGG CAA TCA TCT TCC |
|  |  |  | R: TCA ACT GGC TGT CTG TGT |
|  |  |  |  |
| Elongation of very long chain fatty acids 5 | *elovl5* | AY660879 | F: CCT CCT GGT GCT CT ACA AT |
|  |  |  | R: GTG AGT GTC CTG GCA GTA |
|  |  |  |  |
| Elongation of very long chain fatty acids 6 | *elovl6* | JX975702 | F: GTG CTG CTC TAC TCC TGG TA |
|  |  |  | R: ACG GCA TGG ACC AAG TAG T |
|  |  |  |  |
| Fatty acid desaturase 2 | *fads2* | AY055749 | F: GCA GGC GGA GAG CGA CGG TCT GTT CC |
|  |  |  | R: AGC AGG ATG TGA CCC AGG TGG AGG CAG AAG |
|  |  |  |  |
| Growth hormone receptor-type 1 | *ghr1* | AF438176 | F: ACC TGT CAG CCA CCA CAT GA |
|  |  |  | R: TCG TGC AGA TCT GGG TCG TA |
|  |  |  |  |
| Growth hormone receptor-type 2 | *ghr2* | AY573601 | F: GAG TGA ACC CGG CCT GAC AG |
|  |  |  | R: GCG GTG GTA TCT GAT TCA TGG T |
|  |  |  |  |
| Glutathione peroxidase 1 | *gpx1* | DQ524992 | F: GAA GGT GGA TGT GAA TGG AAA AGA TG |
|  |  |  | R: CTG ACG GGA CTC CAA ATG ATG G |
|  |  |  |  |
| Glutathione peroxidase 4 | *gpx4* | AM977818 | F: TGC GTC TGA TAG GGT CCA CTG TC |
|  |  |  | R: GTC TGC CAG TCC TCT GTC GG |
|  |  |  |  |
| Glucose-regulated protein 170 kDa | *grp170* | JQ308821 | F: CAG AGG AGG CAG ACA GCA AGA C |
|  |  |  | R: TTC TCA GAC TCA GCA TTT CCA GAT TTC |
|  |  |  |  |
| Glucose-regulated protein 94 kDa | *grp94* | JQ308820 | F: AAG GCA CAG GCT TAC CAG ACA G |
|  |  |  | R: CTT CAG CAT CAT CGC CGA CTT TC |
|  |  |  |  |
| Glucose-regulated protein 75 kDa | *grp75* | DQ524993 | F: TCC GGT GTG GAT CTG ACC AAA GAC |
|  |  |  | R: TGT TTA GGC CCA GAA GCA TCC ATG |
|  |  |  |  |
| Fatty acid binding protein, heart | *hfabp* | JQ308834 | F: CTG GGT GTG GGC TTC GCT AC |
|  |  |  | R: CTC TGT GTT CTT GAT GGT GCT CTG |
|  |  |  |  |
| Hypoxia inducible factor 1*α* | *hif1α* | JQ308830 | F: CAG ATG AGC CTC TAA CTT GTG GAC |
|  |  |  | R: TTA GCA AGA ATG GTG GCA AGA TGA G |
|  |  |  |  |
| Hepatic lipase | *hl* | EU254479 | F: TTG TAG AAG GTG AGG AAA ACT G |
|  |  |  | R: GCT CTC CAT CAG ACC ATC C |
|  |  |  |  |
| Insulin-like growth factor 1 | *igf1* | AY996779 | F: TGT CTA GCG CTC TTT CCT TTC A |
|  |  |  | R: AGA GGG TGT GGC TAC AGG AGA TAC |
|  |  |  |  |
| Insulin-like growth factor 2 | *igf2* | AY996778 | F: TGG GAT CGT AGA GGA GTG TTG T |
|  |  |  | R: CTG TAG AGA GGT GGC CGA CA |
|  |  |  |  |
| Insulin-like growth factor binding protein 1a | *igfbp1a* | KM522771 | F: ACA AAC CAA AAC AGT GCG AGT CCT C |
|  |  |  | R: CCG TTC CAA GAG TTC ACA CAC CAG |
|  |  |  |  |
| Insulin-like growth factor binding protein 1b | *igfbp1b* | MH577189 | F: GCC AAA CAG TGT GAG TCA TC |
|  |  |  | R: ATC TTC TTC CCG TTC CAG G |
|  |  |  |  |
| Insulin-like growth factor binding protein 2a | *igfbp2a* | MH577190 | F: CCA GCA AAG AGA CCA CCT |
|  |  |  | R: TCT TCA TCT CCT GCC TGT G |
|  |  |  |  |
| Insulin-like growth factor binding protein 2b | *igfbp2b* | AF377998 | F: AGC GAT GTG TCC TGA GAT AGT GAG |
|  |  |  | R: GCA CCG TGG CGT GTA GAC C |
|  |  |  |  |
| Insulin-like growth factor binding protein 4 | *igfbp4* | KM658998 | F: GGC ATC AAA CAC CCG CAC AC |
|  |  |  | R: ATC CAC GCA CCA GCA CTT CC |
|  |  |  |  |
| Lipoprotein lipase | *lpl* | AY495672 | F: CGT TGC CAA GTT TGT GAC CTG |
|  |  |  | R: AGG GTG TTC TGG TTG TCT GC |
|  |  |  |  |
| Superoxide dismutase [Mn] | *mn-sod / sod2* | JQ308833 | F: CCT GAC CTG ACC TAC GAC TAT GG |
|  |  |  | R: AGT GCC TCC TGA TAT TTC TCC TCT G |
|  |  |  |  |
| NADH-ubiquinone oxidoreductase chain 2 | *nd2* | KC217558 | F: TAG GTT GAA TGA CCA TCG TA |
|  |  |  | R: GGC TAA GGA GTT GAG GTT |
|  |  |  |  |
| NADH-ubiquinone oxidoreductase chain 5 | *nd5* | KC217559 | F: CCT AAA CGC CTG AGC CCT GG |
|  |  |  | R: GCT GTA AAC GAG GTG GCT AGA AGG |
|  |  |  |  |
| Proliferator-activated receptor γ coactivator 1*α* | *pgc1α* | JX975264 | F: CGT GGG ACA GGT GTA ACC AGG ACT C |
|  |  |  | R: ACC AAC CAA GGC AGC ACA CTC TAA TTC T |
|  |  |  |  |
| 85kDa calcium-independent phospholipase A2 | *pla2g6* | JX975708 | F: CGC CAA GGA ACT CGG AAA GAT GCT |
|  |  |  | R: ACC GCA CAG CCA TCA GAG TCT |
|  |  |  |  |
| Peroxisome proliferator-activated receptor α | *pparα* | AY590299 | F: TCT CTT CAG CCC ACC ATC CC |
|  |  |  | R: ATC CCA GCG TGT CGT CTC C |
|  |  |  |  |
| Peroxisomeproliferator-activated receptor γ | *pparγ* | AY590304 | F: CGC CGT GGA CCT GTC AGA GC |
|  |  |  | R: GGA ATG GAT GGA GGA GGA GGA GAT GG |
|  |  |  |  |
| Peroxiredoxin 3 | *prdx3* | GQ252681 | F: ATC AAC ACC CCA CGC AAG ACT G |
|  |  |  | R: ACC GTT TGG ATC AAT GAG GAA CAG ACC |
|  |  |  |  |
| Peroxiredoxin 5 | *prdx5* | GQ252683 | F: GAG CAC GGA ACA GAT GGC AAG G |
|  |  |  | R: TCC ACA TTG ATC TTC TTC ACG ACT CC |
|  |  |  |  |
| Stearoyl-CoA desaturase 1a | *scd1a* | JQ277703 | F: CGG AGG CGG AGG CGT TGG AGA AGA AG |
|  |  |  | R: AGG GAG ACG GCG TAC AGG GCA CCT ATA TG |
|  |  |  |  |
| Stearoyl-CoA desaturase 1b | *scd1b* | JQ277704 | F: GCT CAA TCT CAC CAC CGC CTT CAT AG |
|  |  |  | R: GCT GCC GTC GCC CGT TCT CTG |
|  |  |  |  |
| Sirtuin1 | *sirt1* | KF018666 | F: GGT TCC TAC AGT TTC ATC CAG CAG CAC ATC |
|  |  |  | R: CCT CAG AAT GGT CCT CGG ATC GGT CTC |
|  |  |  |  |
| Sirtuin2 | *sirt2* | KF018667 | F: GAA CAA TCC GAC GAC AGC AGT GAA G |
|  |  |  | R: AGG TTA CGC AGG AAG TCC ATC TCT |
|  |  |  |  |
| Uncoupling protein 1 | *ucp1* | FJ710211 | F: GCA CAC TAC CCA ACA TCA CAA G |
|  |  |  | R: CGC CGA ACG CAG AAA CAA AG |
