# Supplementary material for: From Brewery Waste to Sustainable Aquafeed: Harnessing Nannochloropsis Microalgae for Fishmeal‐Free Gilthead Sea Bream Diets
Source: Aquac Nutr. 2026 May 21;2026:1003936. doi: 10.1155/anu/1003936 (PMC13191778; doi:10.1155/anu/1003936)
Supplement: Supplementary file 4 — Supporting Information 4 Table S4. Primers for qPCR amplification of anterior intestine ( ∗) and head kidney (†) transcripts. [file ANU-2026-1003936-s008.docx]

**Supplementary Table 2**. Primers for qPCR amplification of anterior intestine (*) and head kidney (**^†^**) transcripts.

| **Gene** | **Symbol** | **GenBank** | **Primer** |
| --- | --- | --- | --- |
| Alpha-2-macroglobulin | *a2m***^†^** | AY358020 | F: TCC TGG GTG ACA TTC TGG GT |
|  |  |  | R: CCG TAT GGC ATC CTC AGC AG |
|  |  |  |  |
| Beta-actin | *actb****^†^** | KY388508 | F: TCC TGC GGA ATC CAT GAG A |
|  |  |  | R: GAC GTC GCA CTT CAT GAT GCT |
|  |  |  |  |
| Intestinal-type alkaline phosphatase | *alpi** | KF857309 | F: CCG CTA TGA GTT GGA CCG TGA T |
|  |  |  | R: GCT TTC TCC ACC ATC TCA GTA AGG G |
|  |  |  |  |
| Beta 2 microglobulin | *b2m***^†^** | MF979881 | F: GGC ACT TCC ATC TGA CCA AGA |
|  |  |  | R: GCT GAA CCG CTC TCC ACG |
|  |  |  |  |
| Complement factor C3 | c3**^†^** | HM543456 | F: GCT TAC GCT CTT CTT GCT CTG GTG AA |
|  |  |  | R: CAT CTG ACA ACT GGT CTG GCA TCG T |
|  |  |  |  |
| Caspase 3 | casp3**^†^** | EU722334 | F: GCC AAC GGA CCT GGA CCT G |
|  |  |  | R: CCA TCG CCT CTC CTC GCA TCT A |
|  |  |  |  |
| C-C chemokine receptor type 3 | *ccr3****^†^** | KF857317 | F: CTA CAT CAG CAT CAC CAT ACG CAT CCT |
|  |  |  | R: TGG CAC GGC ACT TCT CCT TCA |
|  |  |  |  |
| C-C chemokine receptor type 9 | *ccr9** | KF857318 | F: TCC CTG AGT TAA TCT TCG CCC AAG TG |
|  |  |  | R: TGT TGT ATT CGT TGT TCC AGT AGA CCA GAG |
|  |  |  |  |
| C-C chemokine receptor type 11 | *ccr11** | KF857319 | F: GCT ACG ATT ACA GTT ATG AA |
|  |  |  | R: TAG ATG ATT GGG AGG AAG |
|  |  |  |  |
| CD209 antigen-like protein D | *cd209d** | KF857327 | F: CGC CAC GAG CAT GAG GAC AA |
|  |  |  | R: TCT TGC CAG AAT CCA TCA CCA TCC A |
|  |  |  |  |
| CD302 antigen | *cd302** | KF857328 | F: GGA CCA GAG GAA GAG CAC ATC |
|  |  |  | R: GAC CAG GGC GGA CAT CAG |
|  |  |  |  |
| Cluster of differentiation 3 zeta chain | *cd3x***^†^** | MF175235 | F: ATG GCG GTC CAG ACG AGG GTT TC |
|  |  |  | R: ACC AGC GAG GAC AGG ACC AGC AG |
|  |  |  |  |
| Cluster of differentiation 4 | *cd4-1****^†^** | AM489485 | F: TCC TCC TCC TCG TCC TCG TT |
|  |  |  | R: GGT GTC TCA TCT TCC GCT GTC T |
|  |  |  |  |
| Cluster of differentiation 8α | *cd8a***^†^** | EU921630 | F: GCA GCA ACG GTA ACA CGA ACG |
|  |  |  | R: CCA GTA TGA GCG GAG TAC AGA ACA |
|  |  |  |  |
| Cluster of differentiation 8*β* | *cd8β****^†^** | KX231275 | F: CCG AAA TGT GGA AGA CTG GAA CTC |
|  |  |  | R: CTT TGG AGG TAA GGT TGG AGG GAT |
|  |  |  |  |
| Cadherin-1 | *cdh1** | KF861995 | F: TGC TCC ATA CAG CGT CAC CTT ACA |
|  |  |  | R: CTC GTT CAT CCT AGC CGT CCA GTT |
|  |  |  |  |
| Cadherin-17 | *cdh17** | KF861996 | F: GAT GCC CGC AAC CCA GAG |
|  |  |  | R: CCG TTG ATT CAC TGC CGT AGA C |
|  |  |  |  |
| C-C chemokine CK8 / C-C motif chemokine 20 | *ck8 / ccl20****^†^** | GU181393 | F: CCG TCC TCA TCT GCT TCA TAC T |
|  |  |  | R: GCT CTG CCG TTG ATG GAA C |
|  |  |  |  |
| Claudin-12 | *cldn12** | KF861992 | F: CTC TCA GGG CTA CAC ATC TAC CTA TGC |
|  |  |  | R: ACA TTC GTG AGC GGC TGG AG |
|  |  |  |  |
| Claudin-15 | *cldn15** | KF861993 | F: CCG ATT GTG GAA GTA GTG GCT CTG GT |
|  |  |  | R: CAG CAT CAC CCA ACC GAC GAA CC |
|  |  |  |  |
| C-type lectin domain family 10 member A | *clec10a***^†^** | KF857329 | F: CGA CTC TGG ACT CCC TCA |
|  |  |  | R: CGT TGT TGA TGG TGC GTT C |
|  |  |  |  |
| Macrophage colony-stimulating factor 1 receptor 1 | *csf1r1****^†^** | AM050293 | F: TTG CGT GTG GTG AGG AAG GAA GGT |
|  |  |  | R: AGC AGG CAG GGC AGC AGG TA |
|  |  |  |  |
| Gap junction Cx32.2 protein | *cx32.2** | KF862000 | F: CGA GGT GTT CTA TCT GCT CTG TA |
|  |  |  | R: CTT GTG GGT GCG AGT CCT |
|  |  |  |  |
| Coxsackievirus and adenovirus receptor homolog | *cxadr** | KF861998 | F: CAT CAG AGG ACT ACG AGA GG |
|  |  |  | R: CAT CTT GGC AGC ATT TGG T |
|  |  |  |  |
| Desmoplakin | *dsp** | KF861999 | F: GCA GAA GGA GCA CGA GAC CATC |
|  |  |  | R: GGG TGT TCT TGT CGC AGG TGA A |
|  |  |  |  |
| Liver type fatty acid-binding protein | *fabp1** | KF857311 | F: GTC CTC GTC AAC ACC TTC ACC AT |
|  |  |  | R: CGC CTT CAT CTT CTC GCC AGT |
|  |  |  |  |
| Intestinal fatty acid-binding protein | *fabp2** | KF857310 | F: CGA GCA CAT TCC GCA CCA AAG |
|  |  |  | R: CCC ACG CAC CCG AGA CTT C |
|  |  |  |  |
| Ileal fatty acid-binding protein | *fabp6** | KF857312 | F: ACC CAG GAC GGC AAT ACC |
|  |  |  | R: CGA CGG TGA AGT TGT TGG T |
|  |  |  |  |
| Fucolectin | *fcl****^†^** | KF857331 | F: CCA TAC TGC TGA ACA GAC CAA CC |
|  |  |  | R: TGA TGG AGG TGA CGA TGT AGG A |
|  |  |  |  |
| Transcription factor HES-1-B | *hes1-b** | KF857344 | F: GCC TGC CGA TAT GAT GGA A |
|  |  |  | R: GGA GTT GTG TTC ATG CTT GC |
|  |  |  |  |
| Immunoglobulin M | *igm****^†^** | JQ811851 | F: ACC TCA GCG TCC TTC AGT GTT TAT GAT GCC |
|  |  |  | R: CAG CGT CGT CGT CAA CAA GCC AAG C |
|  |  |  |  |
| Immunoglobulin T membrane-bound form | *igtm****^†^** | KX599201 | F: AGA CGA TGC CAG TGA AGA GGA TGA GT |
|  |  |  | R: CGA AGG AGG AGG CTG TGG ACC A |
|  |  |  |  |
| Interleukin-1 beta | *il1β****^†^** | AJ419178 | F: GCG ACC TAC CTG CCA CCT ACA CC |
|  |  |  | R: TCG TCC ACC GCC TCC AGA TGC |
|  |  |  |  |
| Interleukin-6 | *il6****^†^** | EU244588 | F: TCT TGA AGG TGG TGC TGG AAG TG |
|  |  |  | R: AAG GAC AAT CTG CTG GAA GTG AGG |
|  |  |  |  |
| Interleukin-7 | *il7****^†^** | JX976618 | F: CTA TCT CTG TCC CTG TCC TGT GA |
|  |  |  | R: TGC GGA TGG TTG CCT TGT AAT |
|  |  |  |  |
| Interleukin-8 | *il8****^†^** | JX976619 | F: CAG CAG AGT CTT CAT CGT CAC TAT TG |
|  |  |  | R: AGG CTC GCT TCA CTG ATG G |
|  |  |  |  |
| Interleukin-10 | *il10****^†^** | JX976621 | F: AAC ATC CTG GGC TTC TAT CTG |
|  |  |  | R: GTG TCC TCC GTC TCA TCT G |
|  |  |  |  |
| Interleukin-12 subunit beta | *il12β****^†^** | JX976624 | F: ATT CCC TGT GTG GTG GCT GCT |
|  |  |  | R: GCT GGC ATC CTG GCA CTG AAT |
|  |  |  |  |
| Interleukin-15 | *il15****^†^** | JX976625 | F: GAG ACC AGC GAG CGA AAG GCA TCC |
|  |  |  | R: GCC AGA ACA GGT TAC AGG TTG ACA GGA A |
|  |  |  |  |
| Interleukin-34 | *il34****^†^** | JX976629 | F: TCT GTC TGC CTG CTG GTA G |
|  |  |  | R: ATG CTG GCT GGT GTC TGG |
|  |  |  |  |
| Krueppel-like factor 4 | *klf4** | KF857346 | F: ACA TCA CCG CAC GCA CAC |
|  |  |  | R: AAC CAC AGC CCT CCC AGT C |
|  |  |  |  |
| Galectin-1 | *lgals1** | KF862003 | F: GTG TGA GGA GGT CCG TGA TG |
|  |  |  | R: ACT GTA GAG CCG TCC GAT AGG |
|  |  |  |  |
| Galectin-8 | *lgals8** | KF862004 | F: GGC GGT GAA CGG CGG TCA |
|  |  |  | R: GCT CCA GCT CCA GTC TGT GTT GAT AC |
|  |  |  |  |
| Macrophage mannose receptor 1 | *mrc1****^†^** | KF857326 | F: CTT CCG ACC GTA CCT GTA CCT ACT CA |
|  |  |  | R: CGA TTC CAG CCT TCC GCA CAC TTA |
|  |  |  |  |
| Mucin 2 | *muc2** | JQ277710 | F: ACG CTT CAG CAA TCG CAC CAT |
|  |  |  | R: CCA CAA CCA CAC TCC TCC ACA T |
|  |  |  |  |
| Mucin 13 | *muc13** | JQ277713 | F: TTC AAA CCC GTG TGG TCC AG |
|  |  |  | R: GCA CAA GCA GAC ATA GTT CGG ATA T |
|  |  |  |  |
| Proliferating cell nuclear antigen | *pcna** | KF857335 | F: CGT ATC TGC CGT GAC CTG T |
|  |  |  | R: AGA ACT TGA CTC CGT CCT TGG |
|  |  |  |  |
| Tight junction protein ZO-1 | *tjp1** | KF861994 | F: AAG CAG TAT TAC GGT GAC TCA |
|  |  |  | R: TGC ATC CCT GGC TTG TAG |
|  |  |  |  |
| Toll-like receptor 2 | *tlr2****^†^** | KF857323 | F: CAT CTG CGA CTC TCC TCT CTT CCT |
|  |  |  | R: ATT CAA CAA TGG AGC GGT GGA CTT |
|  |  |  |  |
| Toll-like receptor 5 | *tlr5****^†^** | KF857324 | F: TCG CCA ATC TGA CGG ACC TGA G |
|  |  |  | R: CAG AAC GCC GAT GTG GTT GTA AGA C |
|  |  |  |  |
| Toll-like receptor 9 | *tlr9****^†^** | AY751797 | F: GCC TTC CTT GTC TGC TCT TTC T |
|  |  |  | R: GCC GTA GAG GTG CTT CAG TAG |
|  |  |  |  |
| Tumor necrosis factor alpha | *tnfα****^†^** | AJ413189 | F: CAG GCG TCG TTC AGA GTC TC |
|  |  |  | R: CTG TGG CTG AGA GGT GTG AG |
|  |  |  |  |
| Zeta-chain-associated protein kinase 70 | *zap70***^†^** | MF175239 | F: TGG TGA AGG AGG AGA TGA TGA GG |
|  |  |  | R: GCG AAC GAT GTA GCG GTT GT |
