# Supplementary figures and images for: From Brewery Waste to Sustainable Aquafeed: Harnessing Nannochloropsis Microalgae for Fishmeal‐Free Gilthead Sea Bream Diets
Source: Aquac Nutr. 2026 May 21;2026:1003936. doi: 10.1155/anu/1003936 (PMC13191778; doi:10.1155/anu/1003936)

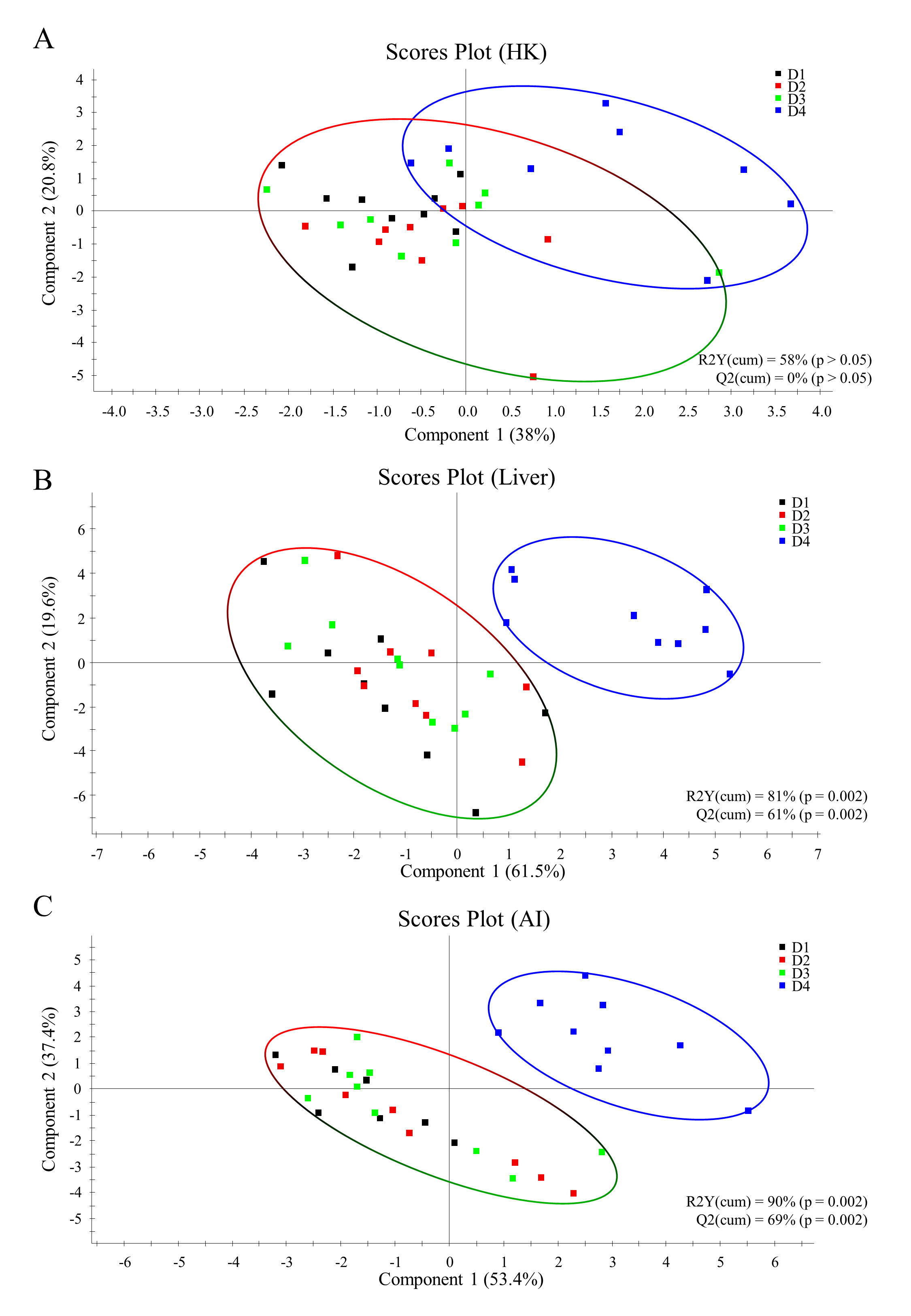

Supplement: Supplementary file 5 — Supporting Information 5 Figure S1. Two‐dimensional PLS‐DA score plots of (A) liver, (B) anterior intestine and (C) head kidney gene expression, representing the distribution of the samples between the first two components in the model. The cumulative explained R2Y(cum) and predicted Q2(cum) variance, as well as the p‐values of the permutation plot can be found at the bottom‐right side of the figure. [file ANU-2026-1003936-s004.tif]

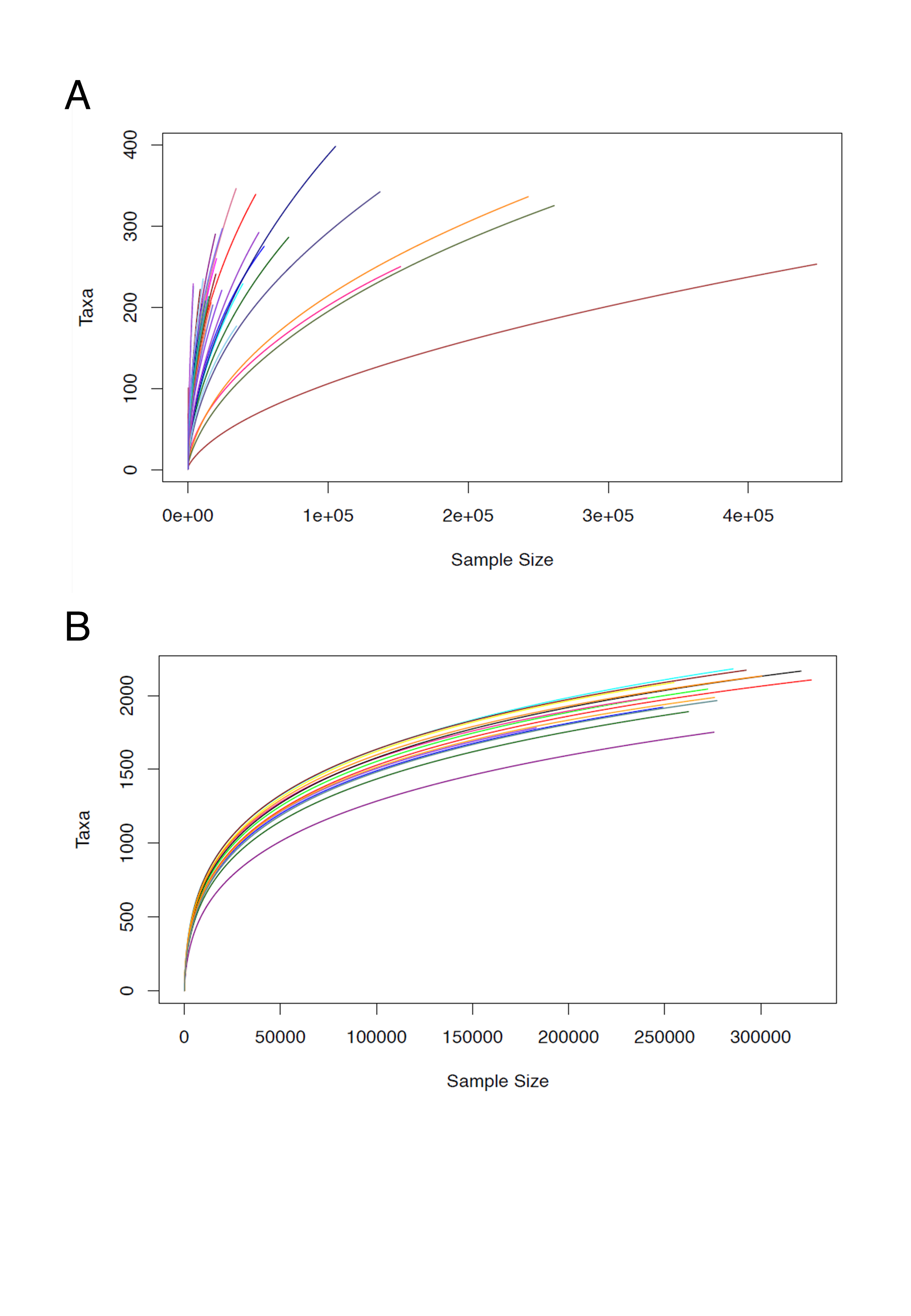

Supplement: Supplementary file 6 — Supporting Information 6 Figure S2. Permutation plots of the PLS‐DA model represented in Figures 1 and 3. [file ANU-2026-1003936-s005.tif]

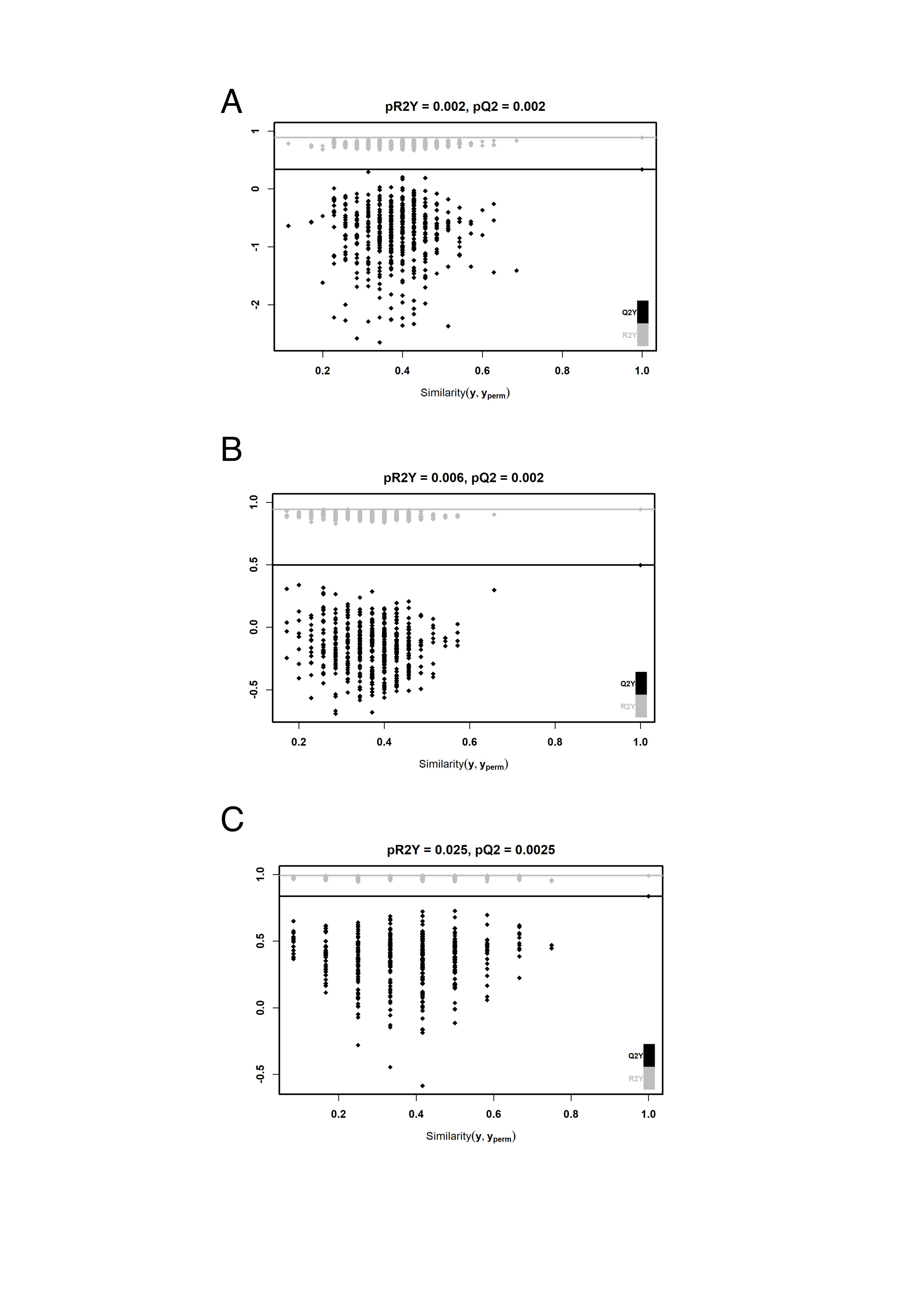

Supplement: Supplementary file 8 — Supporting Information 8 Figure S4. Species richness estimators (Chao1 and ACE) and diversity indexes (Shannon and Simpson) of (A) AI samples from fish fed D1, D2, D3 and D4, and (B) water samples from inlet pipe (W‐INT) or tanks‐containing fish (W‐D1, W‐D2, W‐D3 and W‐D4). [file ANU-2026-1003936-s003.tif]
